# Composite CDE: modeling composite relationships between common data elements for representing complex clinical data

**DOI:** 10.1186/s12911-020-01168-0

**Published:** 2020-07-03

**Authors:** Hye Hyeon Kim, Yu Rang Park, Suehyun Lee, Ju Han Kim

**Affiliations:** 1 Seoul National University Biomedical Informatics (SNUBI), Division of Biomedical Informatics, Seoul National University College of Medicine, Seoul, 03080 Republic of Korea; 2 Department of Biomedical Systems Informatics, Yonsei University College of Medicine, Seoul, 03722 Republic of Korea; 3 Department of Biomedical Informatics, College of Medicine, Konyang University, Daejeon, 35365 Republic of Korea

**Keywords:** Common data elements, Semantic interoperability, Semantic relationship, Metadata registry

## Abstract

**Background:**

Semantic interoperability is essential for improving data quality and sharing. The ISO/IEC 11179 Metadata Registry (MDR) standard has been highlighted as a solution for standardizing and registering clinical data elements (DEs). However, the standard model has both structural and semantic limitations, and the number of DEs continues to increase due to poor term reusability. Semantic types and constraints are lacking for comprehensively describing and evaluating DEs on real-world clinical documents.

**Methods:**

We addressed these limitations by defining three new types of semantic relationship (*dependency*, *composite*, and *variable*) in our previous studies. The present study created new and further extended existing semantic types (*hybrid* atomic and *repeated* and *dictionary* composite common data elements [CDEs]) with four constraints: *ordered*, *operated*, *required*, and *dependent*. For evaluation, we extracted all *atomic* and *composite* CDEs from five major clinical documents from five teaching hospitals in Korea, 14 Fast Healthcare Interoperability Resources (FHIR) resources from FHIR bulk sample data, and MIMIC-III (Medical Information Mart for Intensive Care) demo dataset. Metadata reusability and semantic interoperability in real clinical settings were comprehensively evaluated by applying the CDEs with our extended semantic types and constraints.

**Results:**

All of the CDEs (*n* = 1142) extracted from the 25 clinical documents were successfully integrated with a very high CDE reuse ratio (46.9%) into 586 CDEs (259 *atomic* and 20 unique *composite* CDEs), and all of CDEs (*n* = 238) extracted from the 14 FHIR resources of FHIR bulk sample data were successfully integrated with high CDE reuse ration (59.7%) into 96 CDEs (21 atomic and 28 unique composite CDEs), which improved the semantic integrity and interoperability without any semantic loss. Moreover, the most complex data structures from two CDE projects were successfully encoded with rich semantics and semantic integrity.

**Conclusion:**

MDR-based extended semantic types and constraints can facilitate comprehensive representation of clinical documents with rich semantics, and improved semantic interoperability without semantic loss.

## Background

Data harmonization and interoperability are essential for advancing biomedical research. These features can be achieved by representing clinical data in a standard format, and they are crucial for facilitating understanding and sharing data across diverse translational studies [1, 2]. A data element (DE) is defined as the fundamental unit of data which contains information with a clear conceptualized meaning, together with its representation, and is considered as the correct approach for standardizing data and improving data quality (DQ) and efficiency.

The ISO/IEC 11179 Metadata Registry (MDR) standard describes a method of standardizing and registering DEs to make them understandable and shareable between studies and institutions. MDR-based DE provide data uniformly and interoperability between clinical studies and institutions since they are specified based on a standard metadata model that consists of a sets of attributes, which are delineating the definition, identification, representation, classification, and permissible values [3–5].

The terms DE and common data element (CDE) have been used interchangeably in many ways. However, it can be clearly explained by defining these two terms as the following. The term DE is an atomic unit of data that has precise meaning and precise semantics in metadata. CDE is a data element that is common to multiple data sets across different studies [6]. In this paper, we used the term DE to specifically describe the concept of metadata, but for all other cases we used the term CDE.

CDEs are increasingly being used by clinical researchers in trials for harmonizing data collected across diverse studies. The use of standardized CDEs provides various benefits to investigators including (1) rapid and efficient study start-up by enabling access to defined CDEs and case report forms (CRFs) and (2) enriched data sharing and aggregation using standard definitions and forms [7].

The use of CDEs has been extended to clinical practice by using standardized CDEs for representing the clinical information in electronic health records (EHRs). For example, Newton et al. included phenotype data in EHRs using CDEs to facilitate EHR-driven genomic studies [8]. The National Institutes of Health have developed ISO/IEC 11179 MDR-based CDEs providing a controlled terminology for data descriptors. They also encouraged clinical researchers to use CDEs to facilitate data harmonization [5]. CDEs have been adopted in numerous clinical domains including cancer, stroke, epilepsy, rare disease, emergency medicine, and radiology for patient care and research. Utilizing CDEs will facilitate secondary data use (i.e., ‘collect once and use many times’), which is an approach to data standardization for spanning silos in primary and secondary data use [9].

However, ISO/IEC 11179 MDR focuses only on the representation of individual and independent CDEs without providing the ability to describe constraints for a CDE nor relationships among different CDEs, which are essential for fully describe, semantically compose, and correctly interpret CDEs of clinical documents [10–13]. Although ISO/IEC 11179 MDR standard describes Derived Data Element (DDE) [14] detailing the relationship between a CDE and another CDE from which it is derived with the rule controlling its derivation, this approach is inherently limited by requiring one or more input CDEs and the DDE becoming output DE. For example, while CDEs for describing systolic blood pressure (SBP) and diastolic blood pressure (DBP) can be easily defined as two separate ones annotated with standardized metadata conforming to the ISO/IEC 11179 MDR standard, these two CDEs become mere input CDEs and a separate output CDE should be created as the DDE. Also, a constraint between the two CDEs such as ‘the SBP must be greater than the DBP’ is usually described outside of the CDEs for there is no designated reason for the CDEs to carry constraint information.

To address these challenges in our previous study [10], we proposed three types of semantic relationships (i.e, *variable*, *dependency*, and *composite* relationships) representing semantic constraints or rules among multiple CDEs. These relationships can be described as follows: First, CDEs are in a *variable* relationship when they can be systematically derived from a base CDE by applying a standardized concept from a controlled vocabulary as the variable. For example, the meanings of two CDEs for ‘normal value range of laboratory test, Albumin’ and ‘normal value range of laboratory test, Homocysteine’ are closely related, differing only in the laboratory test names of ‘Albumin’ and ‘Homocysteine.’ It means many lab tests related CDEs can be assigned to one *variable* CDE. The *variable* relationship can systematically represent all these variations as a single CDE, ‘DE: Normal value range of lab test *x*,’ by specifying a controlled vocabulary such as LOINC. The *variable* relationship can therefore systematically reduce the number of required CDEs. Second, a CDE is in a *dependency* relationship may influence the possible determinations of the value space of the CDE(s) base on the value of another CDE(s). For example, the value of a certain CDE may be defined as the sum of the values of a set of CDEs in a questionnaire. Third, the *composite* relationship can be conveniently applied to integrate several interrelated CDEs into a *composite* CDE. For example, the medical history of a patient is likely to be more informative when body parts are correctly assigned, which can be achieved by grouping ‘DE: Body System for Medical History’ and ‘DE: Medical History Specify’ into the *composite* CDE of ‘DE: Medical History.’ However, we realized that our previous work, supports relatively simple semantic relationships among CDEs and is not robust enough to cover many other specific challenges associated with CDEs used in real-world clinical forms.

**Fig. 1:**
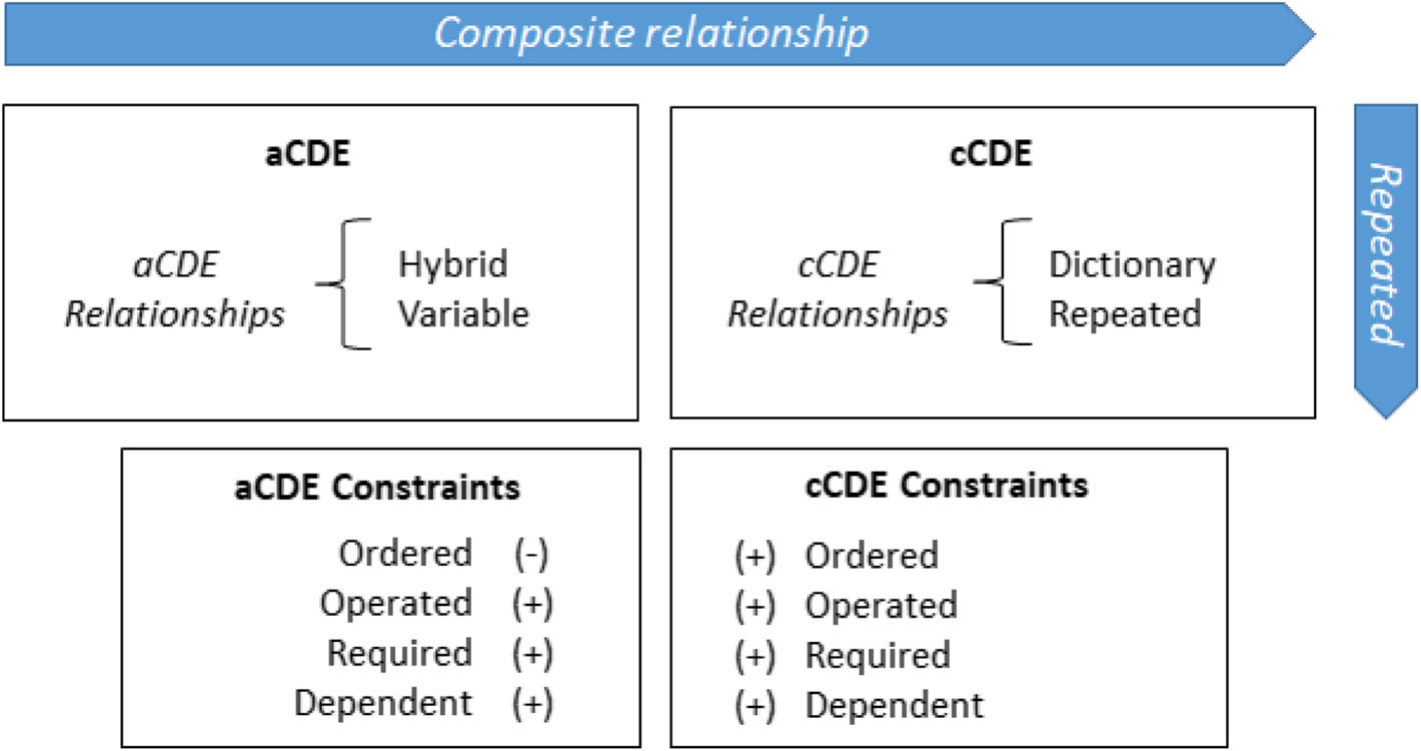
Overview of the formal relationship between aCDE and cCDEs with extended semantic types and CDE-type specific constraints

The present study further proposes extended semantic types (*hybrid* atomic CED (aCDE) and *repeated* and *dictionary* composite CDEs (cCDEs)) and four semantic constraints (*ordered*, *operated*, *required*, and *dependent*) for correctly representing even more complex but essential semantic relationships between CDEs that are found in real-world clinical documents (Fig. 1). We found useful patterns characterizing challenging cases, that required further semantic definitions and descriptions as the following four cases;

### Data entries with multiple data types

A data type determines the type of data that can be entered and stored in a CDE and each CDE contains only one data type [15]. However, we found that free-text-based data entry in many clinical documents stored in EHRs often allows multiple data types to be entered and stored in the same attribute. For example, a laboratory result for syphilis normally has a numeric data type that allows numeric values (e.g., ‘0.8’) as input. However, it often also requires the entry of string or logical data such as ‘negative’ or ‘false’ as input. Sometimes creating two strictly separate CDEs for the same laboratory result for syphilis (i.e., numeric and string) may cause more confusion than not. We found that sometimes it is better to allow either numeric or string data types for the same value domain. We created a value property (*hybrid*) to make it possible to ensure that conventional multiple data types are available in the same CDE by explicitly defining *hybrid* data type for a CDE.

### Dictionary data entries

Data may refer to a controlled biomedical vocabulary for several reasons such as adherence to standards, semantic enrichment for better understanding, and input validation for improving semantic integrity. A CDE referring to a controlled biomedical vocabulary was defined as being in a *variable* relationship in our previous study [10]. We extended the concept of the *variable* relationship to *dictionary data entries* in order to tightly link a set of CDEs via a ‘foreign key’ between a real-world dictionary database and a controlled biomedical vocabulary. This also ensures that a set of CDEs and tuples with rich attributes provided by the dictionary are linked with their proper data type definitions and value domains.

### Tabular data entries with repeated data entry

Clinical data are frequently described in tabular formats. A tabular data entry is an enclosed structure in which a composed set of CDEs is repetitively listed for repeated observations. For example, body weight and height may be measured for each patient when she/he visits for treatment. The set of data items such as body weight, height, and date of measurement should both be collected together and repeatedly. We created a value property (*repeat*) to ensure that the values that belong to the same set of CDEs are identified as such.

### Data constraints

Highly interrelated CDEs in a clinical document need to be defined by semantic constraints for better interchange of semantics and context. By specifying constraints on an aCDE, users can further narrow down the definition of what a valid value really means. For example, a derived value such as BMI (body mass index) can be automatically calculated from the values of the two aCDEs for body weight and height. Because the values of body-weight and height aCDEs should not be null, a *required* constraint should be applied to each of the two aCDEs to make the BMI aCDE to be valid. The calculation formula to obtain BMI is described by an *operated* constraint of BMI aCDE.

For another example, for an aCDE related to the question of whether any drug side effect has happened with permissible answers of ‘Yes’ and ‘No’, the following aCDE, “Specify the drug side effect”, holds only when the value was ‘Yes’. These two aCDEs are in a *dependent* relationship with each other and the sequence of the two has an order. The dependent and sequence relationships can be defined by *dependent* and *ordered* constraints.

## Methods

### Data sources: 2 CDE projects

The National Institute of Neurological Disorders and Stroke (NINDS) CDE Project [16] is an ongoing effort to develop data standards for use in clinical research in neuroscience. It was initiated in 2006 to standardize data collection across neurological-disorder-related clinical studies funded by the NINDS. As of October 2016, the NINDS CDE project included 20 studies with 11,296 distinct CDEs. The NINDS CDEs are not fully compliant with ISO/IEC 11179. Instead they are provided with only simple CDE descriptions and definitions. However, a part of NIND CDEs that are registered in National Cancer Institute (NCI) cancer Data Standards Registry (caDSR) and reviewed by the NCI cancer Biomedical Informatics Grid project manager, conforms fully with the ISO/IEC 11179 MDR standard. In the present study, we used part of the NINDS CDEs, which are 308 (3.1%) stroke and general CDEs of the NINDS in 57 CRFs (Supplementary Tables S1) that are registered in the caDSR. Selected CDEs within the context of their CRFs were explored for challenging cases requiring new semantic relationships that we have defined.

The DialysisNet and Avatar Beans Project is a tablet- and phone-based mobile application developed by the Health Avatar Initiative [17]. The project started in 2013, and it has established clinical data standards for managing and harmonizing hemodialysis data across multiple medical institutions in Korea [18, 19]. This project aims to improve the management of chronic kidney disease and end-stage renal disease by using an integrated mobile application for data collection and documentation. The DialysisNet application was initially built upon 122 distinct hemodialysis-associated CDEs based on CRFs from major four hemodialysis centers (Supplementary Tables S2). We used 11,428 CDEs from the above two projects for comprehensively defining and evaluating new CDE relationships and constraints.

### Designating key concepts

The CRFs and clinical documents from the two CDE projects incorporate all the data collection items with CDEs. We first examined the CDEs to formalize the above mentioned four challenging cases. Figure 1 depics the formal relationships between atomic (aCDE) and composite (cCDE) CDEs with type-specific constraints. Since the core structure of a CDE is a name–value pair augmented by DE concept-domain and value-domain details, an aCDE is a single unambiguously described data item [19]. Our previous and simple-minded definition of cCDE as a set of interrelated aCDEs [9] was extended to include two new semantic relationships: *dictionary* and *repeated* cCDEs.

For example, a drug side effect is regarded as an undesirable secondary effect that occurs in addition to the desired therapeutic effect of a medication. To correctly represent ‘a drugs side effect’, at least three types of information needs to be presented: ‘drug name’, ‘drug dosage’, ‘drug side effect’. One can define the three types of information as aCDEs and then combine them to compose a cCDE.

We extracted aCDEs and cCDEs from the above mentioned two DE projects (NINDS and DialysisNet CDE Projects) and applied the extended semantic types and constraints. We then mapped and integrated the CDEs in order to comprehensively evaluate the metadata reusability and semantic interoperability in the clinical-practice setting.

### Evaluation scheme

For the purpose of evaluating the utility of the newly proposed semantic types and constraints, we used three different data sources: (1) deriving CDEs from clinical documents, (2) Fast Healthcare Interoperability Resources (FHIR) based structured data, and (3) practical clinical dataset from MIMIC-III (Medical Information Mart for Intensive Care).

For deriving CDEs from clinical docments, we collected 25 clinical documents in real-world clinical practice, comprising five documents including *Admission Note*, *Initial Medical Examination Note*, *Discharge Summary*, *Emergency Note*, and *Operation Note* from five major teaching hospitals in Korea: Seoul National University Hospital, Ajou University Medical Center, Pusan National University Hospital, Gachon University Gil Hospital, and Chonnam National University Hospital. It contains Patient, PastHistory, AdmissionInformation, Operation, FamilyHistory, SocialHistory, LabResult, Medication, VitalSign, Treatments, and PhysicalExam [18]. We chose these 25 clinical documents since these documents are used in common by all five hospitals and are essential in the process of patient admission to discharge, for representing the specificity of the data. The limits of these 25 clinical documents are their insufficiency in providing a richness of depth and detail concerning the levels of clinical data. Thus, we added two different structured data from the FHIR bulk sample data and the MIMIC-III demo dataset.

FHIR is propagated as an open standard describing data formats and elements, known as ‘resources’ and an application programming interface (API) for exchanging EHR. FHIR’s clinical resource definitions are concrete, intuitive concepts such as MedicationPrescription, AdverseReaction, Procedure, and Condition. The standard was created by the Health Level Seven International (HL7) healthcare standards organization. We downloaded FHIR bulk sample data, which is exported from a FHIR server to a pre-authorized client by using FHIR bulk Downloader sample app [20–22]. Among 145 resources of FHIR version 4 [23], the FHIR bulk sample data contains 14 resources; AllergyIntolerance, CarePlan, Claim, Condition, Goal, Encounter, Observation, DiagnosticReport, Immunization, MedicationRequest, ImagingStudy, Organization, Patient, and Procedure. Although we could analyze metadata of all FHIR resources through the structural information provided by HL7, it was necessary to review the actual sample data with metadata to confirm the relationships and constraints among the data. Thus, we chose 14 out of the 145 FHIR resources.

The MIMIC-III clinical database contains comprehensive clinical data relating to tens of thousands of Intensive Care Unit patients. MIMIC-III is a large, freely-available database comprising of deidentified health-related data associated with over 40,000 patients who stayed in critical care units of the Beth Israel Deaconess Medical Center between 2001 and 2012. The Dataset has 26 tables which includes vital signs, medications, laboratory measurements, observations and notes charted by care providers, fluid balance, procedure codes, diagnostic codes, imaging reports, hospital length of stay, survival data, and more. We downloaded the MIMIC-III demo dataset that is limited to 100 patients. While the number of patients were limited, the metadata and data-schema were identical [24, 25].

The evaluation process consisted of the following three steps: CDE extraction, CDE integration, and construction of semantic relationships among the CDEs. We counted the numbers of CDEs generated in each step as an evaluation measure of the structural efficiency for the 25 clinical documents and FHIR bulk sample data. However, the MIMIC-III demo dataset is provided as a relational database, containing tables of data relating to patients. A table is a data storage structure which is similar to a spreadsheet: each column contains consistent information (e.g., patient identifiers), and each row contains an instantiation of that information (e.g. a row could contain the integer 340 in the patient identifier column which would imply that the row’s patient identifier is 340) [25]. We manually reviewed the relationships among the columns of each table, whether there were cases which were covered by our proposed CDE relationships and constraints.

## Results

### Overview of all types of semantic relationships

To address the semantic challenges described above, we defined atomic and composite CDEs using newly proposed three semantic types, i.e., *hybrid*, *dictionary*, and *repeated*, and three constraints, i.e., *ordered*, *operated*, and *required*, in addition to the existing two semantic relationship constraints, i.e., *dependent* and *variable* relationships, defined in our previous study. The newly defined *composite* semantic type replaced the old *composite* relationship constraint that we defined previously [10].

Figure 1 depicts atomic and composite CDEs along with their specific relationships and constraints. An aCDE can be constrained using *variable* and *hybrid* relationships by classifying them as *variable* and *hybrid* aCDEs, respectively. The definition of cCDE as a set of interrelated aCDEs in our previous study [10] was extended to include a clear definition, a separate identifier for reuse, and constraints among aCDEs included in a cCDE. A cCDEs can be classified into *dictionary* and *repeated* cCDEs. The *dependent* relationship was the only relationship constraint in our previous study. We extended it to four constraints: *ordered*, *operated*, *required*, and *dependent*. As shown in the left lower panel in Fig. 1, the *ordered* constraint does not apply to an aCDE.

### Data entries with multiple data types: *Hybrid aCDE*

**Fig. 2:**
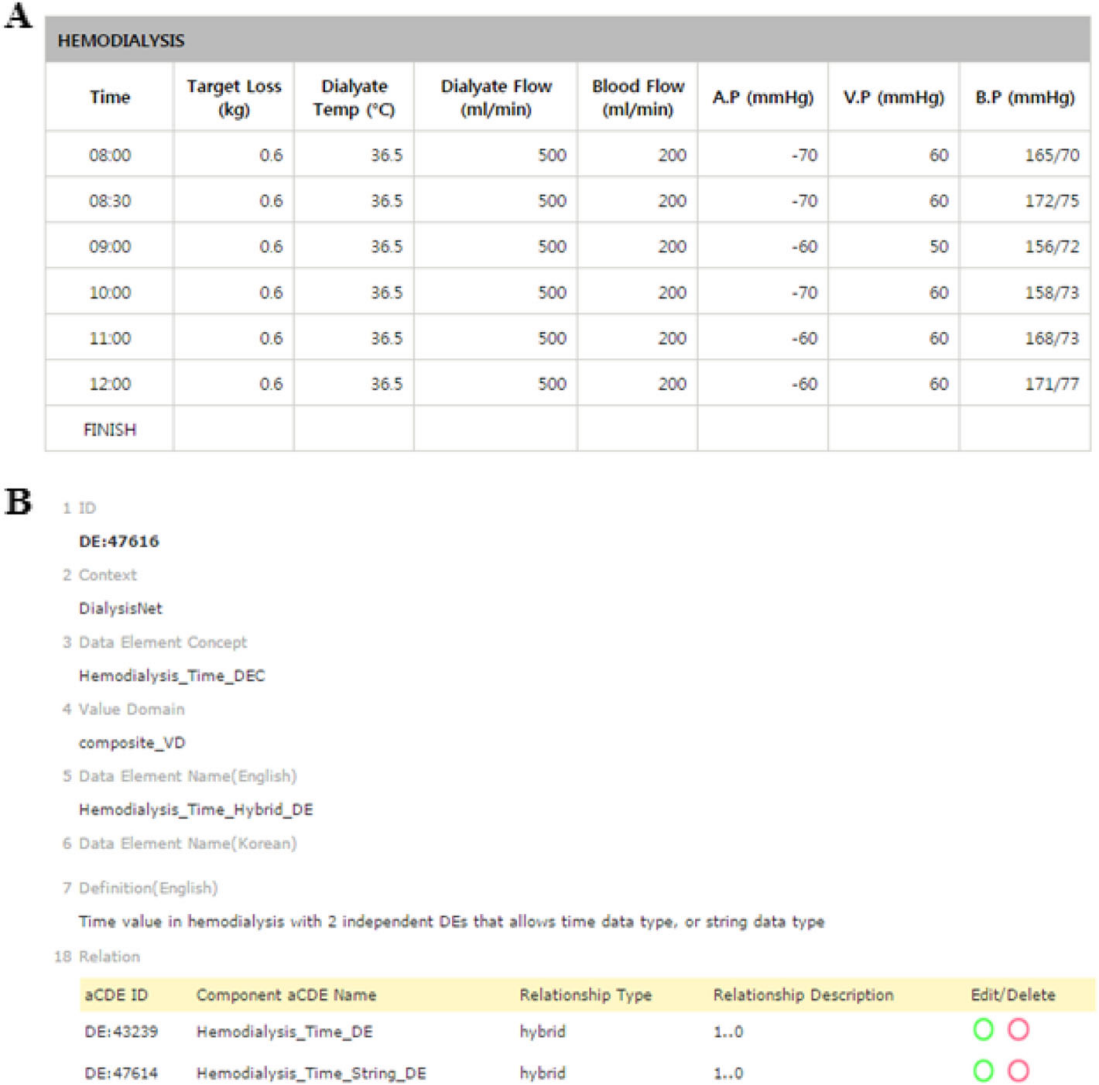
An example hybrid aCDE from a hemodialysis report. **a** The hemodialysis table of the DialysisNet Project has a tabular data-entry format, where *Time* (DE:47616) allows two different data types: time and an enumerated string. **b** The *hybrid* aCDE (DE:47616) contains two aCDEs (DE:43239 and DE:47614) in a hybrid relationship (http://chmr2.snubi.org:8083/chmr/data_element_view.jsp?id=28476)

A *hybrid* aCDE is a particular type of aCDE that allows a value domain with multiple (or hybrid) data types. Technically it includes several aCDEs having the same CDE concept but different value domains. Figure 2a shows a part of a hemodialysis CRF from the DialysisNet and Avatar Beans Project. A time-tagged *hybrid* aCDE was applied to the *Time* attribute in a tabular data-entry format (Fig. 2a). *Time* is defined as a *hybrid* aCDE, Hemodialysis_Time_Hybrid_DE (DE:*47616*). *Time* is derived from two aCDEs, i.e., Hemodialysis_Time_DE (DE:*43239*) and Hemodialysis_Time_String_DE (DE:*47614*) allowing a ‘time’ such as ‘08:00’ and an ‘enumerated-string’ such as ‘Finish’ and ‘Start’, data types, respectively (Fig. 2b). The *hybrid* aCDE *Time* (or Hemodialysis_Time_Hybrid_DE (DE:*47616*)) can capture either a time or an enumerated string value as input.

### Tabular data entries: *Repeated cCDE*

**Fig. 3:**
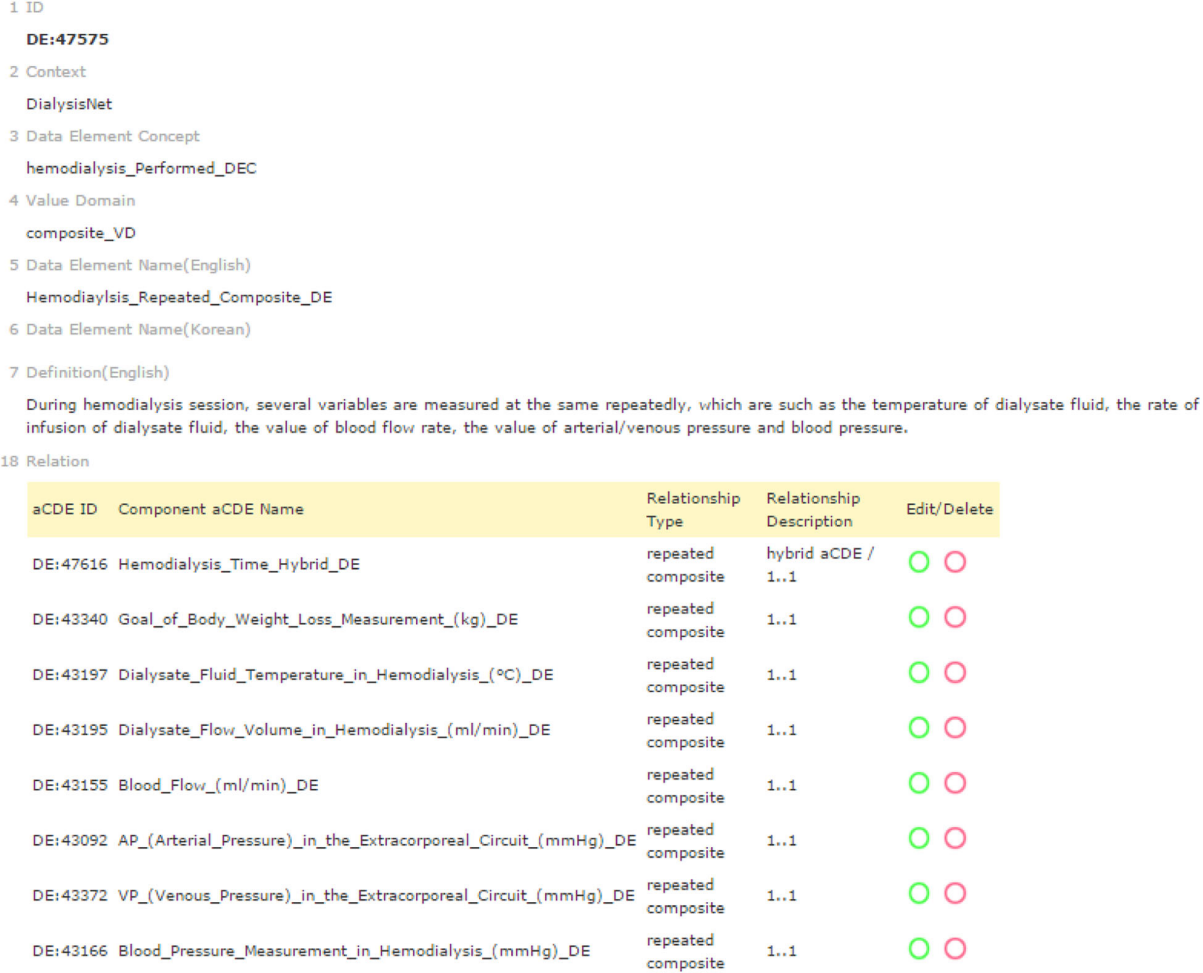
Example of the composition of a repeated cCDE from a hemodialysis report form. A repeated cCDE, ‘DE:47575 Hemodialysis_Repeated_Componsite _DE,’ composed of eight aCDEs from a tabular data-entry format (Fig. 2a) for the DialysisNet hemodialysis project (http://chmr2.snubi.org:8083/chmr/data_element_view.jsp?id=28449)

A *repeated* cCDE is a cCDE that captures data input multiple times in a tabular format. The definition of the *repeated* cCDE prevents the unnecessary creation of redundant CDEs and capture input data in a tabular format. A *repeated* cCDE efficiently captures and displays changes in input values over a certain time span, as shown in Fig. 2a. We first grouped eight aCDEs (i.e., DE:*47616*, DE:*43340*, DE:*43197*, DE:*43195*, DE:*43155*, DE:*43092*, DE:*43372*, and DE:*43166*) to create a cCDE and then assigned *repeated* relationship to create a *repeated* cCDE, Hemodialysis_Repeated_Componsite _DE (DE:*47575*) (Fig. 3). As shown in Fig. 2, DE*:47616* is a *hybrid* aCDE contained in a *repeated* cCDE (DE:*47575*).

### Dictionary data entries: *Dictionary* cCDE

Our previous study [10] defined a *variable* CDE as a CDE that contains a controlled biomedical vocabulary variable. Similarly, a cCDE containing a *variable* aCDE as the primary key of a dictionary table can be defined as a *dictionary* cCDE. This approach provides a way to encode an entire dictionary table as well as a controlled vocabulary into a single *dictionary* cCDE, and thereby capture comprehensive biomedical knowledge from a database. A *dictionary* cCDE provides a useful means to apply relevant attributes of a dictionary database to constrain and validate input values to the *dictionary* cCDE.

**Fig. 4:**
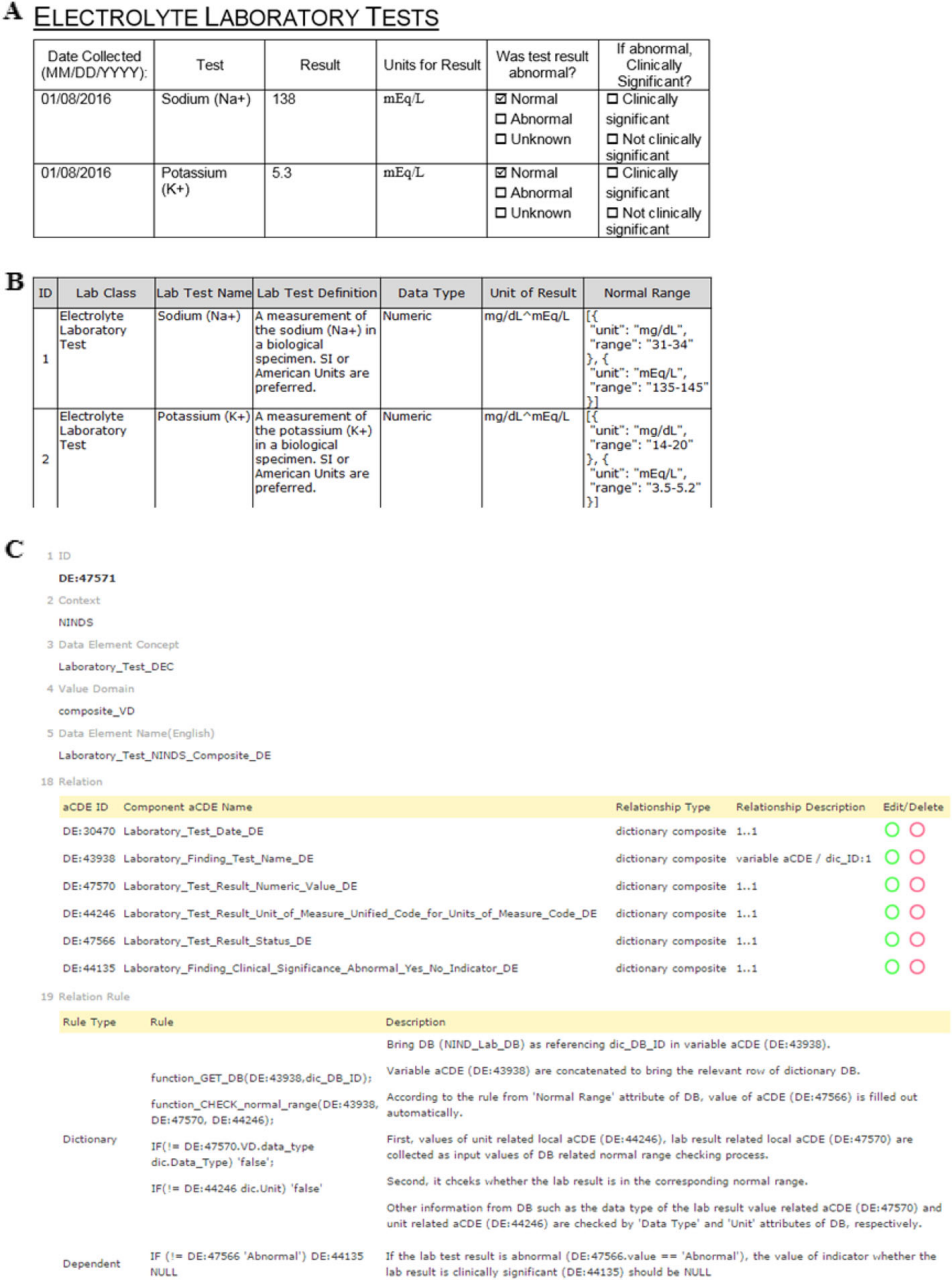
Creation of a dictionary cCDE for a CRF. **a** The ‘Electrolyte Laboratory Tests’ table on a clinical document is provided as an example tabular data-entry document to capture laboratory test results for sodium (Na+) and potassium (K+) along with two clinical evaluation attributes. **b** We constructed the ‘Electrolyte Laboratory Tests Dictionary’ table by extracting the relevant attributes from the CDEs defined in the ‘Recommended Labs for Stroke’ from the NINDS CDE project. **c** The *dictionary* cCDE (DE:47571) consists of six aCDEs that include a variable aCDE (DE:43938) that relates the *dictionary* cCDE to the dictionary table in Fig. 4b. Two rules for clinical evaluation are presented (http://chmr2.snubi.org:8083/chmr/data_element_view.jsp?id=28445)

Figure 4a displays a typical data-entry document for laboratory test results in a tabular format. The ‘Electrolyte Laboratory Tests’ form from ‘Recommended Labs for Stroke’ of the NINDS CDE project [26] consists of six attributes including the laboratory test name, laboratory test result, unit of the laboratory test result, an indicator for whether the laboratory test result was abnormal, and another indicator for whether the laboratory test result was clinically significant when the laboratory test result was abnormal. Figure 4b shows a part of the structured NINDS ‘Electrolyte Laboratory Tests Dictionary’ reference table. The *Unit of Result* attribute supports multiple units that are delimited by ‘^’. The *Normal Range* attribute is also separated according to the *Unit of Result* and is represented in JSON (Javascript object notation)-type encoding.

A dictionary cCDE can systematically capture the entire ‘Electrolyte Laboratory Tests’ data-entry document as ‘DE:*47571* Laboratory_Test_NINDS_Composite_DE,’ which is composed of six aCDEs (Fig. 4c, Relation) that include a *variable* aCDE, ‘DE:*43938* Laboratory_Finding_Test_Name_DE,’ which functions as the foreign key to refer to the primary key for the ‘Lab Test Name’ of the ‘Electrolyte Laboratory Tests Dictionary’ table of Fig. 4b.

Now that the *dictionary* cCDE, DE:*47571*, is successfully related to the NINDS ‘Electrolyte Laboratory Tests’ table via the *variable* aCDE (DE:*43938*), it provides a method to evaluate the validity of input value of 138 mEq/L to *Result* and *Units for Result* for a *Test* [‘Sodium (Na+)’], with respect to the *Normal Range* (i.e., 135 ~ 145 mEq/L) provided by the dictionary table connected via the primary key. The input value to *Was test result abnormal?* can also be automatically evaluated using the biomedical knowledge provided by the dictionary table. Moreover, when the value of *Was test result abnormal?* (DE:*47566*) is ‘Abnormal,’ the value of *If abnormal, Clinically Significant?* (DE:*44135*) can automatically be constrained to contain a value other than null. This constraint can be encoded by a *Dependent Rule*, as shown Relation Rule in Fig. 4c.

Figure 4c Relation Rule shows how a *dictionary* cCDE accompanied by its constraint rules are defined. For the two evaluation cases listed in Fig. 4b, both a *Dictionary Rule* and a *Dependent Rule* are defined by symbolic logic (or pseudocode) with the accompanying *Descriptions*. *Dictionary Rule* defines how to use biomedical knowledge contained in a dictionary table and *Dependent Rule* defines the interrelatedness of aCDEs in a cCDE by using *dependent* constraint relationship

### Semantic restriction: *Constraints*

**Fig. 5:**
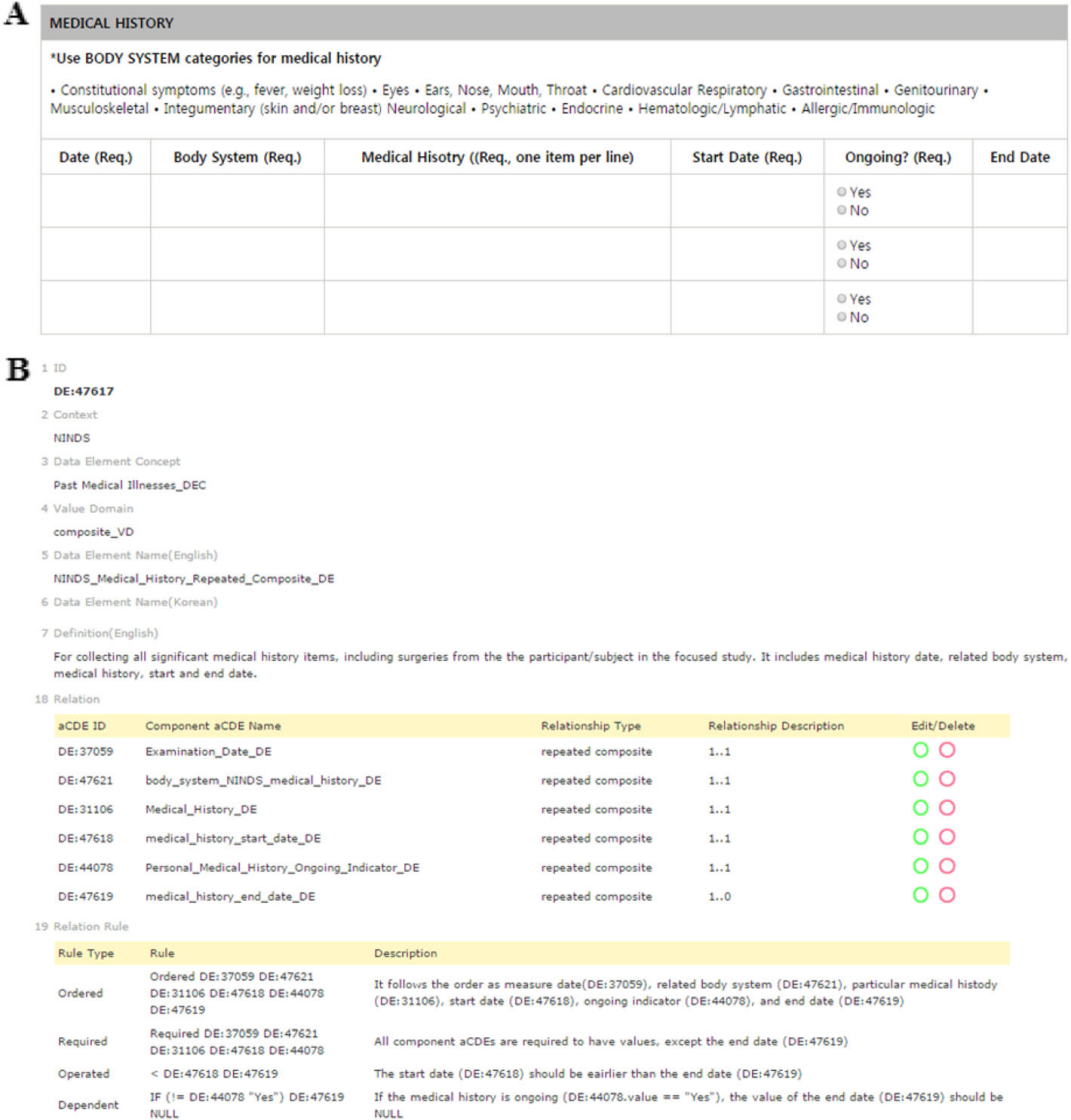
Encoding Operated, Ordered, Required, and Dependent constraints for a repeated cCDE. **a** A ‘Medical History’ clinical document presented in a tabular format containing six attributes. **b** A *repeated* cCDE is created with the corresponding six aCDEs along with four constraint rules: (1) the start date (DE:47618) should be earlier than the end date (DE:47619): (< DE:47618 DE:47619); (2) all attributes are required to have values other than null, except for the end date (DE:47619): (Required DE:37059 DE:47621 DE:31106 DE:47618 DE:44078, 3) when a certain medical history is not ongoing (DE:44078), the end date (DE:47619) cannot be obtained, and vice versa: (IF (! = DE:44078 ‘Yes’) DE:47619 NULL); and (4) aCDEs can be ordered according to a constraint statement such as (Ordered DE:37059 DE:47621 DE:31106 DE:47618 DE:44078 DE:47619) (http://chmr2.snubi.org:8083/chmr/data_element_view.jsp?id=28477)

We defined four constraints that support the creation of a robust clinical document by specifying the interrelationship among many aCDEs. We defined four classes of operators: assignment, arithmetic, logical, and relational. *Order* can only be applied to aCDEs contained in a cCDE. However, the other three constraints (*operated*, *required*, and *dependent*) can be applied to both independent aCDEs and those contained in cCDEs (Fig. 1). We created symbolic logic with prefix notation [27] (Table 1) to describe the order of operations and to formulate constraints. More practical examples are shown in Fig. 5 to demonstrate how constraints are applied to a *repeated* cCDE as well. The four constraints are described as follows:

1. **Operated**. Table 1A presents the standard BMI formula [BMI (in kg/m^2^) = weight / (height × height)] in a prefix notation as (/ CDE30 CDE31 CDE31 100,100), where CDE30 and CDE31 represent *Body Weight Value* in kg and *Body Height Value* in cm, respectively. Both ‘cm’ and ‘m’ units of height measurements are supported by IF conditional statement to manage different units: (IF (= CDE31.unit_of_measure ‘m’) (/ CDE30 CDE31 CDE31) (/ CDE30 CDE31 CDE31 100,100)).
2. **Required**. A *Required* constraint applied to an aCDE means that the aCDE must have a value other than null. Table 1B lists the demographic information of a clinical document, constraining ‘*Patient Age (*CDE40*)’ and ‘*Gender (*CDE41*)’ as *required* by the statement (Required CDE40 CDE41).
3. **Dependent**. It might be necessary to dynamically enable or disable a certain aCDE according to the value(s) of other aCDE(s). For example, a gender-specific CDE might only be applied to subjects of the applicable gender. Table 1C presents an example for checking whether a patient is a current (*CDE20*) or past (*CDE21*) smoker in order to obtain the age when tobacco use was started (*CDE22*). A nonsmoker can conveniently skip *CDE22* if (= CDE20 CDE21 ‘No’) by setting the value of CDE22 as null. In other words, a rule such as (IF (or (! = CDE20 ‘Yes’) (! = CDE21 ‘Yes’)) CDE22 NULL) can be imposed. Another constraint can be imposed to check illogical input values such as (= CDE20 CDE21 ‘Yes’) if necessary.
4. **Ordered**. The ordering of aCDEs (especially in a cCDE) is important for certain conditions and contexts. CDEs in Table 1C can be ordered by a constraint statement such as (Ordered CDE20 CDE21 CDE22).

**Table 1:** Encoding *operated*, *required*, *dependent*, and *ordered* constraints for CDEs with prefix notation. Examples of (A) an *operated* constraint for calculating BMI, (B) a *required* constraint for demography information, (C) a *dependent* constraint for smoking history, and (D) an *ordered* constraint

Constraints	Example of Clinical Documents	Set of CDE IDs and Names
	Prefix Notation for Formulating Constraints	
A) Operated	Weight (kg): Height (cm): BMI (kg/m^2^):	*CDE30* Body Weight Value in kg *CDE31* Body Height Value in cm *CDE32* Body Mass Index Value
	(IF (= CDE31.unit_of_measure ‘m’) (/ CDE30 CDE31 CDE31) (/ CDE30 CDE31 CDE31 100,100)); (/ CDE30 CDE31 CDE31 100,100)	
B) Required	1) *Patient Age: 2) *Gender ☐ Female ☐ Male ☐ Unknown ☐ Unspecified ☐ Not reported 3) Ethnicity: ☐ Hispanic or Latino ☐ Unknown ☐ Not Hispanic or Latino ☐ Not reported	*CDE40* Patient Age *CDE41* Patient Gender *CDE42* Patient Ethnicity
	(Required CDE40 CDE41)	
C) Dependent	Smoking History 1) *Current tobacco use? ☐ Yes ☐ No ☐ Unknown 2) *Past tobacco use? ☐ Yes ☐ No ☐ Unknown 3) Age when tobacco use started (years)? (Skip if Q1 and Q2 are both No)	*CDE20* Current Smoking Indicator *CDE21* Past Smoking Indicator *CDE22* Age When Tobacco Use Started
	(IF (or (!= CDE20 ‘Yes’) (!= CDE21 ‘Yes’)) CDE22 NULL)	
D) Ordered	(Ordered CDE20 CDE21 CDE22)

### Evaluation study

To evaluate the usefulness of our newly extended composite semantic relationships, we applied them to CDEs which were systematically extracted from the 25 clinical documents of five teaching hospitals in Korea and from FHIR bulk sample data. At first, we focused on deriving CDEs from clinical documents, which provided many explicit cases that clearly demonstrated the relationships between CDEs. We then wanted to prove that our proposed relationships and constraints were valid in structured clinical dataset as well. It was why we chose two difference types of source data: unstructured and structured data. The evaluation process consisted of the following steps: CDE extraction, CDE integration by using the newly proposed atomic and composite CDEs with semantic enrichments. We examined how the number of CDEs had been reduced from CDE extraction to CDE integration, measuring the structural and semantic efficiency of CDEs for clinical data elements.

Although HL7 FHIR supports mainly structured data, it also provides a document related resource, FHIR Questionnaire*.* To see whether our proposed semantic types can cover FHIR Questionnaire*,* we matched elements of the FHIR Questionnaire resource to our developed relationships and constraints for further evaluation.

For evaluating derived CDEs from clinical documents, we first extracted 84, 48, 70, 83, and 37 CDEs from the five clinical documents, *Admission Note*, *Initial Medical Examination*, *Discharge Summary*, Emergency Note, and *Operation Note*, respectively, of Hospital A. We found that 95 (29.5%) of the 322 aCDEs were reused in at least two of the five clinical documents, resulting in 227 unique aCDEs. We then created clinically relevant cCDEs and applied semantic relationships to them. Of the 84 aCDEs extracted from the *Admission Note* of Hospital A, 55 were successfully captured by 10 cCDEs. Finally, 16 cCDEs successfully captured 110 (48.5%) of the 227 unique CDEs such that 133 (=16 + 117) CDEs (41.3%) were sufficient to represent the initial 322 CDEs extracted from the five clinical documents of Hospital A (Table 2).

**Table 2:** Numbers of aCDEs and cCDEs extracted from five clinical documents used at five teaching hospitals in Korea

Hospital		Admission Note	Initial Medical Examination Note	Discharge Summary	Emergency Not	Operation Note	Total No. of CDEs	^f^ No. of Unique CDEs	^g^ CDE Reuse Rate
A	^a^ CDE	84	48	70	83	37	322	227	29.5%
	^b^cCDE ^c^ (aCDE)	10 (55)	9 (40)	6 (34)	6 (45)	2 (10)	33 (184)	16 (110)	
	^d^ aCDE	29	8	36	38	27	138	117	
	^e^ cCDE + aCDE	39	17	42	44	29	171	133	24.5%
C	CDE	30	35	20	27	26	138	87	37.0%
	cCDE (aCDE)	2 (14)	3 (20)	2 (11)	3 (15)	1 (5)	11 (65)	5 (35)	
	aCDE	16	15	9	12	21	73	52	
	cCDE + aCDE	18	18	11	15	22	84	57	33.3%
G	CDE	70	28	44	54	11	207	161	22.2%
	cCDE (aCDE)	4 (23)	3 (17)	2 (11)	2 (17)	1 (5)	12 (73)	7 (50)	
	aCDE	47	11	33	37	6	134	111	
	cCDE + aCDE	51	14	35	39	7	146	118	18.8%
P	CDE	204	123	46	43	12	428	266	37.9%
	cCDE (aCDE)	7 (177)	4 (99)	3 (34)	3 (39)	0 (0)	15 (349)	7 (177)	
	aCDE	27	24	12	4	12	79	89	
	cCDE + aCDE	34	28	15	7	12	94	96	36.2%
S	CDE	12	6	9	10	10	47	31	34.0%
	cCDE (aCDE)	1 (3)	0	0	1 (4)	0	2 (7)	1 (4)	
	aCDE	9	6	9	6	10	40	27	
	cCDE + aCDE	10	6	9	7	10	42	28	31.9%
Total	CDE	400	240	189	217	96	1142	606	53.1%
	Unique CDE	297	162	142	178	57	836	586	29.9%
	cCDE (aCDE)	15 (224)	14 (152)	9 (71)	9 (90)	2 (10)	49 (547)	20 (327)	
	aCDE	73	10	71	88	47	289	259	
	cCDE + aCDE	88	24	80	97	49	338	279	46.9%

^a^ No. of CDEs extracted from each clinical document from each hospital

^b^ No. of cCDEs created for each clinical document

^c^ No. of aCDEs contained in ^b^cCDEs

^d^ No. of remaining aCDEs that are not contained in any of the cCDEs in each clinical document

^e^ Total no. of CDEs consisting of ^b^cCDEs and ^d^aCDEs that are not contained in any of the cCDEs in each clinical document

^f^ No. of unique CDEs across the five clinical documents

^g^ Reuse ratio of CDEs across the five clinical documents

In the CDE extraction step, we found that applying CDE is an effective way to reduce redundant CDEs (22.2 ~ 37.9%) at each hospital. This means that there were many CDEs shared across the five different clinical documents at each hospital. We found that an even higher CDE reduction rate of 48.7% was achieved by integrating the information for all five hospitals, which indicated that various CDEs were commonly used across five different teaching hospitals.

The CDE integration step involved integrating aCDEs into clinically relevant cCDEs to further structure the clinical documents and then integrating the cCDEs across different clinical documents. For example, when a vital sign-related cCDE contained three aCDEs (‘body weight,’ ‘body temperature,’ and ‘blood pressure’) and another vital sign-related cCDE contained an additional aCDE (‘description the reason of unstable vital sign’), we integrated them into a vital-sign cCDE comprising four aCDEs. The application of these three steps constantly decreased the number of CDEs. Supplementary Tables S3–S5 list the cCDEs and how they were distributed in each document at each hospital. These tables also provide a detailed view of how the 20 unique cCDEs comprised 327 sub-aCDEs. The integrated CDEs not only reduced the number of CDEs with a reuse ratio of up to 46.9% [=(1142–20 – 586)/1142] (Table 2) but also showed greatly improved semantic accuracy and interoperability, which was also supported by the review of the documents by the authors.

We found that the compositions of the clinical documents differed quite markedly across the five hospitals. The clinical documents at Hospitals P and S contained the largest (*n* = 266) and smallest (*n* = 31) numbers of independent CDEs, respectively. We also found that even the same clinical documents showed huge variations in CDE numbers. The number of CDEs in *Admission Notes* varied from 12 at Hospital S to 204 at Hospital P. Hospital P also had the largest number of aCDEs for *Initial Medical Examination Note* (*n* = 123) while Hospital A had the largest number of aCDEs for *Emergency Note* (*n* = 83) and *Operation Note* (*n* = 37).

We also applied constraint rules for the five clinical documents of the five hospitals (Table 3). We could not determine if a CDE was a *hybrid* aCDE partly due to the lack of sufficient input values and partly due to poor descriptions of the response values for the clinical documents. We designated the cCDEs as *basic* cCDEs to distinguish them from *repeated* and *dictionary* cCDEs. A cCDE was on average reused twice among the five documents by the hospitals. We also found that the clinical documents at Hospital A were the best structured and contained the greatest detail with more cCDEs and constraint rules compared to the documents of the other hospitals.

**Table 3:** Numbers of aCDEs, cCDEs, and constraints at five teaching hospitals in Korea

Hospital: CDE Semantic Type		A	C	G	P	S
aCDE	Hybrid	0	0	0	0	0
	Variable	5	2	2	3	0
cCDE	Basic	9 (20)	2 (6)	3 (8)	2 (2)	0
	Repeated	2 (5)	1 (2)	2 (2)	2 (6)	1 (2)
	Dictionary	5 (10)	2 (3)	2 (2)	3 (8)	0
Constraints	Operated	4 (9)	1 (5)	2 (5)	1 (1)	0
	Required	10 (25)	3 (8)	5 (11)	3 (11)	0
	Dependent	15 (26)	0	3 (8)	3 (10)	1 (2)
	Ordered	11 (29)	4 (10)	5 (11)	3 (12)	1 (2)

The numbers before the parentheses represent unique counts

We evaluated the DE relationships and constraints with the same method applied to different data sources, which were 14 FHIR resources from FHIR bulk sample data. We first extracted 238 CDEs and found 142 CDEs (59.7%) were reused in at least 2 of 14 FHIR resources, resulting in 96 unique aCDEs. We then created clinically relevant cCDEs and applied semantic relationships to them. 48 cCDEs successfully captured 194 (81.5%) of 238 CDEs. Finally, 28 cCDEs successfully captured 75 of the 96 unique CDEs such that 49 (=28 + 21) CDEs were enough to represent the initial 238 CDEs extracted from 14 FHIR resources (Table 4). Supplementary Tables S6–S7 list the cCDEs and how they were distributed in each FHIR resources. The fact that more than half of the CDEs has been reused shows that the FHIR data are relatively well standardized and structured. Half of the FHIR resources, i.e., AllergyIntolerance, Condition, Encounter, Goal, MedicationRequest, Organization, and Procedure, were represented by *repeated* cCDEs, which means all extracted CDEs of each FHIR resource became a component aCDEs of the *repeated* cCDEs. These structured data have been reused frequently among different FHIR resources.

**Table 4:** Numbers of atomic and composite CDEs extracted from 14 FHIR resources of FHIR bulk sample data

#	FHIR Resource	^a^CDE	^b^cCDE ^c^ (aCDE)	^d^ aCDE	^e^ cCDE + aCDE
1	AllergyIntolerance	13	2 (13)	0	2
2	CarePlan	18	4 (15)	3	7
3	Claim	21	5 (13)	6	11
4	Condition	13	2 (13)	0	2
5	DiagnosticReport	13	3 (9)	4	7
6	Encounter	15	4 (15)	0	4
7	Goal	4	1 (4)	0	1
8	ImagingStudy	23	3 (14)	11	14
9	Immunization	12	1 (4)	8	9
10	MedicationRequest	14	3 (14)	0	3
11	Observation	22	5 (18)	4	9
12	Organization	15	4 (15)	0	4
13	Patient	42	8 (29)	8	16
14	Procedure	13	3 (13)	0	3
^f^ Total No. of CDEs		238	48 (194)	44	92
^g^ No. of unique CDEs		96	28 (75)	21	49

^a^ No. of CDEs extracted from each FHIR resource sample data

^b^ No. of cCDEs created for each FHIR resource sample data

^c^ No. of aCDEs contained in ^b^cCDEs

^d^ No. of remaining aCDEs that are not contained in any of the cCDEs in each FHIR resource sample data

^e^ Total no. of CDEs consisting of ^b^cCDEs and ^d^aCDEs that are not contained in any of the cCDEs in each FHIR resource sample data

^f^ Total no. of CDEs across 14 FHIR resources

^g^ Total no. of unique CDEs across 14 FHIR resources

While we were mapping our proposed semantic types and constraints to FHIR resources, we found that *hybrid* aCDE and *operated,* and *dependent* constraints were not applicable in FHIR resources. For the case of *hybrid* aCDE, although only one datatype is allowed for each data in FHIR specification, we foun no restriction on the datatype in the FHIR bulk sample data since the data was represented by JSON, and XML. While the *required* and *ordered* constraints were explicitly indicated, *operated,* and *dependent* constraints were not valid in FHIR resources because the rule by which two or more data values were related could not be applied (Table 5).

**Table 5:** Numbers of atomic and composite CDEs and constraints in FHIR bulk data and MIMIC-III demo data

Data Source: CDE Semantic Type		FHIR	MIMIC-III
aCDE	Hybrid	N/A	4
	Variable	3	4
cCDE	General	18 (64)	4 (12)
	Repeated	7 (87)	26 (180)
	Dictionary	3 (17)	4 (17)
Constraints	Operated	N/A	2
	Required	34	52
	Dependent	N/A	N/A
	Ordered	2	N/A

**Fig. 6:**
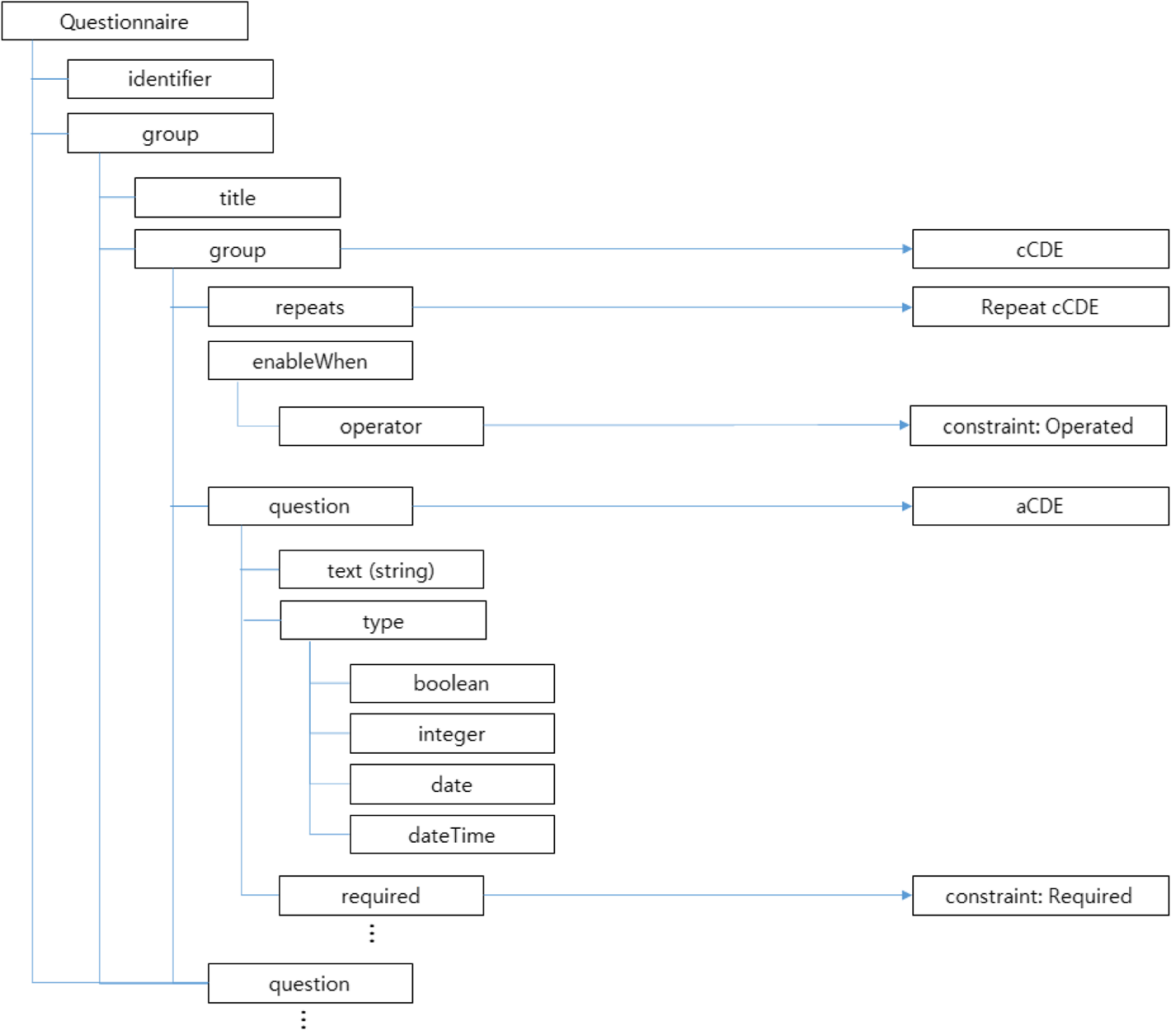
Mapping result of the FHIR Questionnaire resource mapped to the proposed CDE relationships and constraints

Another evaluation was the mapping between our semantic types and constraints to document-associated FHIR resource, Questionnaire*.* Figure 6 represents the mapping of the FHIR structure in extracts on the left side, linked via arrows to the corresponding developed CDE relationships and constraints. The relevant elements in the FHIR Questionnaire resource were *group* and *question*, which represents composite and atomic CDEs (the data model of a single question). Among our three CDE relationships and four constraints, the *repeated* cCDE relationship and the *required* and *operated* constraints were straightforwardly mapped. The FHIR Questionnaire resource is to define both collection forms, surveys and other structures that can be filled out with their context. It had a certain structure to represent relationships among CDEs but value related constraints could not be modelled. For instance, it could not be represented whether the value allows for multiple data types (*Hybrid* aCDE) or whether one value can be changed depending upon another element’s value (Constraint: *Dependent*).

For evaluations with a real dataset, we analyzed 26 tables of the MIMIC-III demo database. These tables were divided into three categories which were classified by different data characteristics: (1) 14 tables for hospital data, (2) three tables for online definitions, and (3) 19 tables for care-value and meta-version ICU related data (Supplementary Tables S8). We first manually reviewed the relationships among the columns of each table. The evaluation process was conducted only for cases in which a relationship was found through the following steps: CDE extraction, CDE integration by using atomic and composite CDEs and then the construction of semantic relationships among the CDEs.

We found four *hybrid* aCDEs that allows numeric data and text data. For example, *VALUE* in LABEVENTS allows for string data and numeric data. If this value is numeric, then VALUENUM represents the same data in a numeric format with an appropriate unit from VALUEUOM for its usability in calculations. The four general cCDEs in Table 5 list cCDEs that includes the *hybrid* aCDE. We also found three *variable* aCDEs associated with its particular *dictionary* cCDE. For example, ICD9_CODE in DIAGNOSES_ICD is matched to the same value as ICD9_CODE in D_ICD_DIAGNOSES. And each table became a *repeated* cCDE because it is composed of a set of related items. All tables have a *required* constraint, and two tables have an *operated* constraint. As MIMIC data is provided as a relational database, *dependent* and/or *ordered* constraints are not applicable. Relational table treats the value of each column independently without ordering based on set inclusion theory (Table 5). Supplementary Tables S8–S9 lists specific results which the MIMIC-III metadata matched to our proposed relationships and constraints.

## Discussion

### Comparison with related studies

Standardization of clinical data using CDEs based on ISO/IEC 11179 metadata standard is clearly one of most effective ways to harmonize data collected from various clinical institutions and studies. The advantages of this approach are (1) providing a consistent data collection tool, (2) improving study data quality, and (3) reducing the cost of data entry and cleansing by having uniform data. However, the limitation of ISO/IEC 11179 of not providing a data structure for representing interrelationships among CDEs has resulted in a gap between the development of CDEs and their utilization in clinical forms. Although ISO/IEC 11179 provides DDEs to overcome the limitation by enhancing interrelated CDEs. A DDE is a DE whose values are derived through a transformation of the values of one or more source CDEs. For example, the DDE of the ‘length of stay in a hospital’ is derived from two independent DEs that counts the number of days from two input CDEs: ‘Admission date’ and ‘Discharge date.’ However, this strategy is far from enough to cover all use cases of interrelated CDEs that we have describe in the Background section.

Table 6 compares the DDE and our CDE semantic relationships. The value of a DDE is derived from input DE(s). Our CDE semantic relationship provides rich semantics for creating atomic and composite CDEs that feature *repeat* and *dictionary* properties, supporting references to outside biomedical resources as described in Table 6. The relatively simple-minded concept of the DDE may be insufficient to cover various CDE semantic relationships since a DDE covers only two constraints: *Operated* and *Ordered*.

**Table 6:** Differences between DDE and our CDE semantic relationships

CDE Semantic Type		Characteristic	Difference from a DDE
aCDE	Hybrid	Allowing the entry of multiple data types in a hybrid aCDE requires aCDEs that support different data types for the same data item	A DDE does not support the entry of multiple types of data
	Variable	Connecting to an outside dictionary database	No dictionary-associated constraint in a DDE
cCDE	General	Containing a set of aCDEs	Do not have output DE(s), but a DDE can be a cCDE
	Repeated	Allowing sequential data entry into a *repeated* cCDE	No *repeated* property in a DDE
	Dictionary	Bringing biomedical knowledge from an outside dictionary database to a *dictionary* cCDE containing a *variable* aCDE as a foreign key to the dictionary table with the *repeated* property	No dictionary connection allowed for a DDE
Constraint	Operated	Allowing mathematical/algebraic expressions between related aCDEs	A DDE has this constraint with the ^a^ CALCULATION type
	Required	Forcing aCDE to have a value other than null	No *required* constraint in a DDE
	Dependent	Dynamic enabling and disabling of an aCDE via a predicate	No *dependent* constraint in a DDE
	Ordered	Ordering a set of aCDEs	A DDE has this constraint by default

^a^ CALCULATION type in DDE only covers arithmetic operators (i.e., +, −, *, /) but, the operated constraints include not only arithmetic operators but also logical operators (i.e., <, >)

There have also been efforts to address the issues of interrelated CDE(s) by applying external data models. The CDISC (clinical data interchange standards consortium) ODM (operational data model), which is an XML-based standardized data model that supports the acquisition and exchange of metadata specifically related to clinical studies, can also be used to overcome the limitations of ISO/IEC 11179. However, it is not sufficiently comprehensive to generate CRFs by importing elements directly [28, 29]. Lin et al. also suggested to use the openEHR approach for modeling CDEs [30]. Though this approach provides a comprehensive structure with two-level modeling, several limitations when implementing openEHRs have been identified in various studies such as immaturity of archetype modification operations, insufficient support for hierarchical archetypes due to their granularity [31, 32], and the cost burden of development and adoption due to the complexity of defining openEHRs. Therefore, instead of utilizing external data models, we propose improving and extending the existing composite relationship by specifying two subtypes of aCDE, three subtypes of cCDEs, and four constraints to take advantage of utilizing CDEs and related technologies.

The newly released version of HL7 FHIR provides the ElementDefinition type, which is the core of the FHIR metadata layer and is closely (conceptually) aligned to ISO/IEC 11179. It has the result of mapping to the other standards as well to help implementers and clinical researchers understand the content and use it correctly. However, they found that the principles from both standards were totally different. FHIR does not differentiate the difference between a CDE and a CDE value and the FHIR specification is heavily type dependent. For instance, HL7 FHIR provides the pair of Questionnaire and QuestionnaireResponse resources and a pair of Appointment and AppointmentResponse resources at the same time. Also, the FHIR specification includes constraints and other concerns that are outside the scope of ISO/IEC 11179. Thus, the HL7 admitted that there still was a shortage of connection between HL7 FHIR and ISO/IEC 11179. It is said that the FHIR Infrastructure work group is considering rolling the DataElement resource into the StructureDefinition resource. If this is done, DataElement resource will be treated as a type of logical model (whether there will be a distinct ‘type’ for it is unclear) [33].

Since the FHIR specification includes concepts for the group and constraints, they were matched with our proposed concepts of *composite* and the part of constraints (ordered, operated). However, some of the semantic types and constraints that we have proposed are not provided by FHIR. We detailed whether our proposed semantic types and constraints were covered by FHIR. Since the FHIR Questionnaire is the only resource, which is related to clinical forms or documents, we distinguished from the other FHIR resources (Table 7).

**Table 7:** Comparison of our proposed semantic types and constraints with the FHIR Questionnaire resource and the other FHIR resources

CDE Semantic Type		FHIR Questionnaire	FHIR other resources
aCDE	Hybrid	No, it does not support the entry of multiple types of data.	Not applicable, there is no restriction on the datatype as it is represented JSON, XML.
	Variable	Yes, it is supported by “coding”.	Yes, it is supported by “coding”.
cCDE	General	Yes, it is supported because the FHIR is following a structured model.	Yes, it is supported because the FHIR is following a structured model.
	Repeated	Yes, it is supported by “repeats”.	Yes, it is supported because the FHIR is allowing repeated representation of the group of items.
	Dictionary	Not applicable, it does not support any value related rule.	Not applicable, it does not support any value related rule.
Constraint	Operated	Allowing only logical operations.	Only resources that have the “operator” are supported (e.g., Observation Resouce).
	Required	Yes, it is supported by “required”.	Yes, it is supported by “required”.
	Dependent	Not applicable, it does not support any value related rule.	Not applicable, it does not support any value related rule
	Ordered	Although not explicit, it is included in the structure.	Only resources that have “sequences” are supported (e.g., Claim Resouce)

### Overcoming the challenges of understanding semantic relationships of form-lEVeL data

This paper has presented an in-depth evaluation of the ISO/IEC 11179 MDR standard based CDE semantic interrelationships in the context of formalizing clinical document structures. For converting form-level data into DE-level data, two cCDEs (*repeated* and *dictionary* cCDEs) and their related constraints were developed, which provide the following benefits:

1. **Repeated cCDEs support clinical data management in a tabular format in a clinical document.** Since multiple value sets are supported to be represented in a unified tabular format, a *repeated* cCDE is useful for managing sequential data entry in a tabular format and for analyzing how the values change over time. A *repeated* cCDE enables standard MDR-based CDE-level descriptions and evaluations of clinical data entry in a tabular format.
2. **Dictionary cCDEs enable biomedical knowledge to be brought from a dictionary database via a variable aCDE.** Data items referencing a certain standard terminology appear frequently on clinical forms. A *dictionary* cCDE can help to include rich semantics from externally managed biomedical terminologies and/or dictionaries, with rich attributes being applied for input data validation.
3. **Four different types of constraints enable rich evaluations of input values.** A prefix notation with functional logic programming can be applied for evaluating user-defined constraints in order to ensure contextual correctness and interrelationships among data items on clinical document.

### Advantages of using CDEs and CDE relationships for building clinical documents

The data element is the atomic unit of data and is associated with a data element concept (DEC, an abstract unit of knowledge for representing semantics) and a value domain (representation of data including the data type and permissible values) according to the ISO/IEC 11179 MDR standard. The DEC is the combination of an object class (a set of entities) and a property (a peculiarity common to all member of an object class). As these two components of DEC are matched to the standard medical terminologies, it strengthens the semantic part. It is an advantage to use CDE. Our proposed new semantic types and constraints comply with this part in the ISO/IEC 11179 standard.

As verified in the evaluation part of this study, building clinical documents with CDEs can provide three major advantages. First, it prevents the generation of redundant data by facilitating predefined and registered CDEs to the MDR. Second, it ensures semantic data integrity since an MDR-based CDE has comprehensive and standardized metadata attributes for data description and the proposed cCDE provides a means to encode rich constraints for inter-CDE relationships. The health data of a patient that are fragmented, dispersed, and duplicated in a variety of clinical documents across different medical centers should be integrated, and mapping data items to CDEs facilitates data integration and semantic interoperability across different clinical documents. Third, clinical data exchange and sharing can be greatly facilitated by this approach.

### Limitation and future work

The real-life clinical documents provide reasonable examples of reality, but particular instances of reality do not necessarily always provide good representative examples. For instance, we found that the quality of data in the clinical documents is dependent on whether the clinicians who wrote these documents were well trained in terminology representation to be inclusive in writing correctly and sufficiently valid clinical documents. If the document provides poor examples, then the outcome of the evaluation will also be poor. It is not only the problem of clinical documents but also it can be applied to when a clinical researcher creates data in the FHIR model or a physician inputs clinical data in an EHR system. Thus, we should measure the DQ, which is one of the aspects of the interoperability that reveals the process of standardizing EHRs to ensure the selected clinical documents are a good representation of the evaluation.

We also found that one essential issue was whether our proposed semantic types and constraints ensure semantical consistency with the use of standard biomedical terminologies. For the instance of data transfer and the purpose of interoperability, it is important to examine how well our proposed semantic types and constraints correspond to the standard biomedical terminologies and how we can address the issue of terminology variations. Although the DEC part of the ISO/IEC 11179 is matched to the standard medical terminologies, when multiple standard biomedical vocabularies are used in the complicated CDEs the above issue can occur. A similar issue can occur when we utilize the dictionary cCDE, since it includes a biomedical vocabulary. For instance, the dictionary cCDE can consider different ‘versions’ of a particular laboratory test with different time stamps, which could end with a differing variance of normal ranges. In other words, even if we reference the same standard vocabulary for the *dictionary* cCDE, the result could be different. We will measure another DQ for semantical consistency from the two issues mentioned above as a future work.

To measure DQ, we will consider the 5 different dimensions of DQ such as completeness, correctness, concordance, plausibility, and currency. The strategies used to assess the dimensions of DQ fell into seven DQ methods such as gold standard, data element agreement, element presence, data source agreement, distribution comparison, and validity check as a future work [34].

## Conclusion

The sharing and understanding of data from multiple different domains can be facilitated by standardization. An MDR-based CDE is considered a type of standardized data with specified concept and value domains. However, ISO/IEC 11179 MDR-based CDEs do not provide the ability to describe constraints on a CDE or relationships among different CDEs, instead merely focusing on single independent CDEs, which makes it difficult to either correctly compose or interpret CDEs on clinical documents. We developed MDR-based extended semantic types and constraints, and it can facilitate comprehensive representation of clinical documents with rich semantics and improved semantic interoperability.

## Supplementary information

**Additional file 1: Supplementary Table S1.** List of NINDS CRFs with stroke CDEs related CRFs (*n* = 35), general CDEs related CRFs (*n* = 15), and common CRFs (*n* = 7). **Supplementary Table S2.** List of DialysisNet related Forms. **Supplementary Table S3.** List of general, dictionary, and repeated cCDEs with the numbers of operated, required, dependent, and ordered constraints extracted from five clinical documents used at five teaching hospitals in Korea. **Supplementary Table S4.** Distribution of aCDEs and cCDEs extracted from five clinical forms used at five teaching hospitals in Korea. **Supplementary Table S5**. List of 327 aCDEs comprising 20 cCDEs. The order of the cCDEs is identical to that in Supplementary Table S1. **Supplementary Table S6** Distribution of aCDEs and cCDEs extracted from 14 FHIR resources of FHIR bulk sample data. List of unique 75 aCDEs comprising by 28 cCDEs from 238 aCDEs. The absence of *repeated* cCDE for some FHIR resources means that the configuration of aCDEs has been changed for each data. **Supplementary Table S7.** List of 75 aCDEs comprising by 28 cCDEs. The order of the cCDEs is identical to that in Supplementary Table S4. **Supplementary Table S8** List of categorized MIMIC-III database in which matched by aCDE, cCDE and constraints. Six tables are related *hybrid* and *variable* aCDE (23%), four tables are related *dictionary* cCDE (15%), and all tables are related to *required* constraints. **Supplementary Table S9** List the detail elements of MIMIC-III database, which were matched to our proposed semantic types and constraints. *Hybrid* aCDEs in four tables, *variable* aCDE in four tables, *operated* constraint in two tables.

## Data Availability

Not Applicable.

## Abbreviations

aCDE: Atomic CDE
BMI: Body mass index
cCDE: Composite CDE
CDE: Common data element
CDISC: Clinical data interchange standards consortium
CRF: Case report form
DDE: Derived data element
DE: Data element
DQ: Data Quality
EHR: Electronic health record
FHIR: Fast Healthcare Interoperability Resources
JSON: Javascript object notation
MDR: Metadata registry
MIMIC-III: Medical Information Mart for Intensive Care
NINDS: National institute of neurological disorders and stroke
ODM: Operational data model

## References

[1] Richesson RL, Krischer J. Data standards in clinical research: gaps, overlaps, challenges and future directions. J Am Med Inform Assoc. 2007. 10.1197/jamia.M2470. 10.1197/jamia.M2470 PMC2213488 17712081

[2] Ferranti JM, Musser RC, Kawamoto K, Hammond WE. The clinical document architecture and the continuity of care record: a critical analysis. J Am Med Inform Assoc. 2006. 10.1197/jamia.M1963. 10.1197/jamia.M1963 PMC1513652 16501180

[3] Mohanty SK, Mistry AT, Amin W, et al. The development and deployment of common data elements for tissue banks for translational research in cancer–an emerging standard based approach for the mesothelioma virtual tissue Bank. BMC Cancer. 2008. 10.1186/1471-2407-8-91. 10.1186/1471-2407-8-91 PMC2329649 18397527

[4] Groft SC, Rubinstein YR. New and evolving rare diseases research programs at the National Institutes of Health. Public Health Genomics. 2013. 10.1159/000355929. 10.1159/000355929 24503586

[5] NIH Common Data Element (CDE) Repository Website. https://www.nlm.nih.gov/cde/. Accessed 20 Mar 2020.

[6] DE and CDE definition in NIM. Website. https://www.nlm.nih.gov/cde/glossary.html#cdedefinition. Accessed 12 Mar 2020.

[7] Saver JL, Warach S, Janis S, et al. Standardizing the structure of stroke clinical and epidemiologic research data: the National Institute of Neurological Disorders and Stroke (NINDS) stroke common data element (CDE) project. Stroke. 2012. 10.1161/STROKEAHA.111.634352. 10.1161/STROKEAHA.111.634352 PMC3493110 22308239

[8] Newton KM, Peissig PL, Kho AN, Bielinski SJ, Berg RL, Choudhary V, Basford M, Chute CG, Kullo IJ, Li R, Pacheco JA, Rasmussen LV, Spangler L, Denny JC. Validation of electronic medical record-based phenotyping algorithms: results and lessons learned from the eMERGE network. J Am Med Inform Assoc. 2013. 10.1136/amiajnl-2012-000896. 10.1136/amiajnl-2012-000896 PMC3715338 23531748

[9] Nahm M, Walden A, McCourt B, et al. Standardising clinical data elements. Int J Funct Inform Personal Med. 2010. 10.1504/IJFIPM.2010.040213.

[10] Park YR, Yoon YJ, Kim HH, Kim JH (2013). Establishing semantic interoperability of biomedical metadata registries using extended semantic relationships. Stud Health Technol Inform.

[11] Nadkarni PM, Brandt CA (2006). The common data elements for cancer research: remarks on functions and structure. Methods Inf Med.

[12] Richesson RL, Nadkarni P. Data standards for clinical research data collection forms: current status and challenges. J Am Med Inform Assoc. 2011. 10.1136/amiajnl-2011-000107. 10.1136/amiajnl-2011-000107 PMC3078665 21486890

[13] ISO/IEC 11179. International Standard, International Electrotechnical Commission, Information technology — Metadata registries (MDR) — Part 3: Registry metamodel and basic attributes. https://webstore.iec.ch/preview/info_isoiec11179-3%7Bed3.0%7Den.pdf, Publication date April 10, 2006.

[14] NCI caDSR Wiki, CDE Curation Tool User Guide- Creating Derived Data Element. Website. https://wiki.nci.nih.gov/display/caDSR/10+-+Creating+Derived+Data+Elements/. Accessed 20 Mar 2020.

[15] Data type in Wikipedia. Website. https://en.wikipedia.org/wiki/Data_type/. Accessed 12 Mar 2020.

[16] NINDS Common Data Elements Website. https://commondataelements.ninds.nih.gov/. Accessed 12 Mar 2020.

[17] Ku HS, Kim S, Kim H, Kim JH. DialysisNet: application for integrating and management data sources of hemodialysis information by continuity of care record. Healthc Inform Res. 2014. 10.4258/hir.2014.20.2.145. 10.4258/hir.2014.20.2.145 PMC4030058 24872913

[18] Park YR, Kim H, An EY, et al. Establishing semantic interoperability in the course of clinical document exchange using international standard for metadata registry. J Korean Med Assoc. 2012. 10.5124/jkma.2012.55.8.729.

[19] Kim JH. Health avatar: an informatics platform for personal and private big data. Healthc Inform Res. 2014. 10.4258/hir.2014.20.1.1. 10.4258/hir.2014.20.1.1 PMC3950259 24627812

[20] Braunstein ML. Healthcare in the age of interoperability: the promise of fast healthcare interoperability resources. IEEE Pulse. 2018. 10.1109/MPUL.2018.2869317. 10.1109/MPUL.2018.2869317 30452344

[21] Braunstein ML. Health Care in the age of interoperability part 6: the future of FHIR. IEEE Pulse. 2019. 10.1109/MPULS.2019.2922575. 10.1109/MPULS.2019.2922575 31395530

[22] FHIR Bulk downloader sample app. Website. https://bulk-data.smarthealthit.org/sample-app/index.html. Accessed Mar. 20, 2020.

[23] HL7 FHIR version 4.0 Resource List. Website. https://www.hl7.org/fhir/resourcelist.html. Accessed Mar. 20, 2020.

[24] Johnson A, Pollard T, Mark R. MIMIC-III Clinical Database Demo (version 1.4). *PhysioNet*. 2019; 10.13026/C2HM2Q.

[25] MIMIC-III Critical Care Database. Website. https://mimic.physionet.org/about/mimic/. Accessed Mar. 20, 2020.

[26] NINDS Common Data Elements. Website. https://www.commondataelements.ninds.nih.gov/Doc/Stroke/F1168_Laboratory_Tests_Permissible_Values_for_Stroke.xlsx. Accessed Mar. 20, 2020.

[27] Wikipedia. Website. https://en.wikipedia.org/wiki/Polish_notation. Accessed Mar. 20, 2020.

[28] Ngouongo SM, Löbe M, Stausberg J. The ISO/IEC 11179 norm for metadata registries: does it cover healthcare standards in empirical research? J Biomed Inform. 2013. 10.1016/j.jbi.2012.11.008. 10.1016/j.jbi.2012.11.008 23246614

[29] Iberson-Hurst D (2004). The CDISC operational data model: ready to roll?. Appl Clin Trials.

[30] Lin CH, Fann YC, Liou DM. An exploratory study using an openEHR 2-level modeling approach to represent common data elements. J Am Med Inform Assoc. 2016. 10.1093/jamia/ocv137. 10.1093/jamia/ocv137 PMC6375118 26911823

[31] Garde S, Hovenga E, Buck J, Knaup P. Expressing clinical data sets with openEHR archetypes: a solid basis for ubiquitous computing. Int J Med Inform. 2007. 10.1016/j.ijmedinf.2007.02.004. 10.1016/j.ijmedinf.2007.02.004 17392019

[32] Späth MB, Grimson J. Applying the archetype approach to the database of a biobank information management system. Int J Med Inform. 2011. 10.1016/j.ijmedinf.2010.11.002. 10.1016/j.ijmedinf.2010.11.002 21131230

[33] HL7 DataElement resource. Website. https://hl7.org/fhir/STU3/dataelement.html. Accessed Mar. 20, 2020.

[34] Weiskopf NG, Weng C. Methods and dimensions of electronic health record data quality assessment: enabling reuse for clinical research. J Am Med Inform Assoc. 2013. 10.1136/amiajnl-2011-000681. 10.1136/amiajnl-2011-000681 PMC3555312 22733976

