# Supplementary material for: Composite CDE: modeling composite relationships between common data elements for representing complex clinical data
Source: BMC Med Inform Decis Mak. 2020 Jul 3;20:147. doi: 10.1186/s12911-020-01168-0 (PMC7333279; doi:10.1186/s12911-020-01168-0)
Supplement: Supplementary file 1 — Additional file 1: Supplementary Table S1. List of NINDS CRFs with stroke CDEs related CRFs (n = 35), general CDEs related CRFs (n = 15), and common CRFs (n = 7). Supplementary Table S2. List of DialysisNet related Forms. Supplementary Table S3. List of general, dictionary, and repeated cCDEs with the numbers of operated, required, dependent, and ordered constraints extracted from five clinical documents used at five teaching hospitals in Korea. Supplementary Table S4. Distribution of aCDEs and cCDEs extracted from five clinical forms used at five teaching hospitals in Korea. Supplementary Table S5. List of 327 aCDEs comprising 20 cCDEs. The order of the cCDEs is identical to that in Supplementary Table S1. Supplementary Table S6 Distribution of aCDEs and cCDEs extracted from 14 FHIR resources of FHIR bulk sample data. List of unique 75 aCDEs comprising by 28 cCDEs from 238 aCDEs. The absence of repeated cCDE for some FHIR resources means that the configuration of aCDEs has been changed for each data. Supplementary Table S7. List of 75 aCDEs comprising by 28 cCDEs. The order of the cCDEs is identical to that in Supplementary Table S4. Supplementary Table S8 List of categorized MIMIC-III database in which matched by aCDE, cCDE and constraints. Six tables are related hybrid and variable aCDE (23%), four tables are related dictionary cCDE (15%), and all tables are related to required constraints. Supplementary Table S9 List the detail elements of MIMIC-III database, which were matched to our proposed semantic types and constraints. Hybrid aCDEs in four tables, variable aCDE in four tables, operated constraint in two tables. [file 12911_2020_1168_MOESM1_ESM.docx]

**Supplementary Table S1. List of NINDS CRFs with stroke CDEs related CRFs (n=35), general CDEs related CRFs (n=15), and common CRFs (n=7).**

| # | Type | FormID | Form Name |
| --- | --- | --- | --- |
| 1 | Common | F0016 | Demographics |
| 2 | Common | F0039 | Electrocardiogram_(ECG) |
| 3 | Common | F0011 | Family_History |
| 4 | Common | F1807 | General_Core_CDES |
| 5 | Common | F0013 | Medical_History |
| 6 | Common | F0020 | Prior_and_Concomitant_Medications |
| 7 | Common | F0026 | Vital_Signs |
| 8 | General | F0021 | Death_Report |
| 9 | General | F1122 | Device_Revision_Replacement_Log |
| 10 | General | F1121 | Devices_Log |
| 11 | General | F0012 | Inclusion_and_Exclusion_Criteria |
| 12 | General | F0038 | Informed_Consent_and_Enrollment |
| 13 | General | F0025 | Laboratory_Tests_and_Tracking |
| 14 | General | F0005 | Participant_Subject_Contact_Information |
| 15 | General | F0037 | Physical_Exam |
| 16 | General | F0004 | Serious_Adverse_Events |
| 17 | General | F0017 | Study_Discontinuation_Completion |
| 18 | General | F0022 | Study_Drug_Compliance |
| 19 | General | F0024 | Study_Drug_Dosing |
| 20 | General | F0299 | Study_Therapies_Compliance |
| 21 | General | F1123 | Surgery_and_Pathology |
| 22 | General | F0008 | Visit_Schedule |
| 23 | Stroke | F1178 | Antithrombotics_and_Risk_ |
| 24 | Stroke | F1154 | Behavioral_History |
| 25 | Stroke | F1170 | Biomarker_Guidelines |
| 26 | Stroke | F1169 | Biospecimen_Collection_and_Processing |
| 27 | Stroke | F1156 | History_Data_Source_and_Reliability |
| 28 | Stroke | F1159 | Hospital_Arrival_Admission |
| 29 | Stroke | F1163 | Hospital_Discharge |
| 30 | Stroke | F1167 | Laboratory_Tests |
| 31 | Stroke | F1168 | Laboratory_Tests_Permissible_Values_for_Stroke |
| 32 | Stroke | F1181 | Lifestyle_Modification_Therapies |
| 33 | Stroke | F1183 | Palliative_Comfort_Care_and_End_of_Life_Issues |
| 34 | Stroke | F1171 | Parenchymal_Imaging |
| 35 | Stroke | F1172 | Perfusion_and_Penumbral_Imaging |
| 36 | Stroke | F1164 | Physical_Neurological_Exam |
| 37 | Stroke | F1155 | Pregnancy_and_Perinatal_History |
| 38 | Stroke | F1158 | Pre-Hospital_Emergency_Medical_Service_(EMS)_Course |
| 39 | Stroke | F1157 | Prior_Functional_Status |
| 40 | Stroke | F1182 | Rehabilitation_Therapies |
| 41 | Stroke | F1151 | Social_Status |
| 42 | Stroke | F1160 | Stroke_Symptoms_Comorbid_Events |
| 43 | Stroke | F1162 | Stroke_Types_and_Subtypes |
| 44 | Stroke | F1187 | Summary_of_Recommended_Measures_for_Activities_of_Daily_Living_ |
| 45 | Stroke | F1202 | Summary_of_Recommended_Measures_for_Clinical_Event_End_Points |
| 46 | Stroke | F1190 | Summary_of_Recommended_Measures_for_Emotional_and_Cognitive_Status |
| 47 | Stroke | F1185 | Summary_of_Recommended_Measures_for_Neurological_Impairment |
| 48 | Stroke | F1193 | Summary_of_Recommended_Measures_for_Participation__Quality_of_Life |
| 49 | Stroke | F1198 | Summary_of_Recommended_Measures_for_Pediatrics |
| 50 | Stroke | F1196 | Summary_of_Recommended_Measures_for_Performance |
| 51 | Stroke | F1184 | Summary_of_Stroke_CDE_Recommendations_by_Outcome_Domain_and_Classification |
| 52 | Stroke | F1180 | Surgical_and_Procedural_Interventions |
| 53 | Stroke | F1179 | Thrombolytic_Reperfusion_Therapies |
| 54 | Stroke | F1173 | Vessel_Carotid_Ultrasound |
| 55 | Stroke | F1174 | Vessel_Imaging_Angiography |
| 56 | Stroke | F1175 | Vessel_Imaging_Transcranial_Color-Coded_Real-time_Sonography_(TCCS) |
| 57 | Stroke | F1176 | Vessel_Imaging_Transcranial_Doppler_(TCD) |

**Supplementary Table S2. List of DialysisNet related Forms.**

| # | Form Name | Contents |
| --- | --- | --- |
| 1 | Individual patient | Demography |
| 2 | Individual patient | Vital signs |
| 3 | Individual patient | Functional status |
| 4 | Individual patient | Medication |
| 5 | Hemodialysis | Dialysis |
| 6 | Hemodialysis | Lab results |
| 7 | Hemodialysis | Dialysis outcomes |
| 8 | Consultation and transfer forms |  |
| 9 | Reasoning helping data |  |

**Supplementary Table S3. List of *general*, *dictionary*, and *repeated* cCDEs with the numbers of *operated*, *required*, *dependent*, and *ordered* constraints extracted from five clinical documents used at five teaching hospitals in Korea.**

| ID | cCDE Name | Reuse Count^a^ | No. of Constraints | | | |  |
| --- | --- | --- | --- | --- | --- | --- | --- |
|  |  |  | *Operated* | *Required* | *Dependent* | *Ordered* | |
| 1 | Patient_Demography_Composite_DE | 15 | 1 | 1 | 0 | 1 | |
| 2 | Past_History_Allergy_Dictionary_Composite_DE | 4 | 0 | 1 | 1 | 1 | |
| 3 | Admission_and_Operation_Composite_DE | 3 | 0 | 1 | 1 | 1 | |
| 4 | Chief_Complaint_Repeated_Composite_DE | 10 | 0 | 1 | 0 | 1 | |
| 5 | Pain_Composite_DE | 3 | 0 | 1 | 1 | 1 | |
| 6 | Medicine_List_Composite_DE | 3 | 0 | 1 | 1 | 1 | |
| 7 | BMI_Composite_DE | 2 | 1 | 0 | 0 | 0 | |
| 8 | Patient_Evaluation_Composite_DE | 1 | 0 | 1 | 3 | 1 | |
| 9 | Past_History_Dictionary_Composite_DE | 3 | 0 | 0 | 0 | 0 | |
| 10 | Patient_Vital_Sign_Repeated_Composite_DE | 5 | 1 | 0 | 0 | 0 | |
| 11 | Current_Medication_List_Dictionary_Composite_DE | 1 | 0 | 1 | 1 | 1 | |
| 12 | Examination_Finding_Dictionary_Composite_D | 3 | 0 | 0 | 0 | 1 | |
| 13 | Treatment_Finding_Composite_DE | 1 | 0 | 1 | 3 | 1 | |
| 14 | Discharge_Medication_Dictionary_Composite_DE | 1 | 0 | 0 | 1 | 0 | |
| 15 | Person_Weight_Change_Composite_DE | 2 | 1 | 0 | 1 | 1 | |
| 16 | Social_History_Composite_DE | 6 | 0 | 1 | 2 | 1 | |
| 17 | Transfer_Discharge_Composite_DE | 3 | 0 | 1 | 2 | 1 | |
| 18 | General_System_Dictionary_Composite_DE | 1 | 0 | 0 | 0 | 0 | |
| 19 | Physical_Exam_Dictionary_Composite_DE | 3 | 0 | 1 | 0 | 1 | |
| 20 | Lab_Result_Dictionary_Composite_DE | 2 | 0 | 0 | 1 | 0 | |

**^a^**The number of reuses in five clinical documents used at five teaching hospitals.

**Supplementary Table S4. Distribution of aCDEs and cCDEs extracted from five clinical forms used at five teaching hospitals in Korea.**

| Hospital | **ID^a^** | **Admission**  **Notes** | **Initial**  **Medical Examination**  **Notes** | **Discharge**  **Notes** | **Emergency Notes** | **Operation**  **Notes** | **Total**  **No. of**  **CDEs** | **^d^No. of Unique**  **CDEs** |
| --- | --- | --- | --- | --- | --- | --- | --- | --- |
| A |  | **10 (55)** | **9 (40)** | **6 (34)** | **6 (45)** | **2 (10)** | **33 (184)** | **16 (110)** |
|  | 1 | 1 (6) | 1 (6) | 1 (6) | 1 (6) | 1 (6) |  | 1 (6) |
|  | 2 | 1 (4) | 1 (3) |  | 1 (3) | 1 (4) |  | 1 (4) |
|  | 3 | 1 (4) | 1 (5) |  | 1 (3) |  |  | 1 (5) |
|  | 4 | 1 (4) | 01 (4) | 1 (5) |  |  |  | 1 (5) |
|  | 5 | 1 (9) | 1 (9) |  |  |  |  | 1 (9) |
|  | 6 | 1 (3) | 1 (3) |  |  |  |  | 1 (3) |
|  | 7 | 1 (3) | 1 (3) |  |  |  |  | 1 (3) |
|  | 8 | 1 (15) |  |  |  |  |  | 1 (15) |
|  | 9 | 1 (3) | 1 (3) | 1 (3) |  |  |  | 1 (3) |
|  | 10 | 1 (4) | 1 (4) |  |  |  |  | 1 (4) |
|  | 11 |  |  |  | 1 (4) |  |  | 1 (4) |
|  | 12 |  |  |  | 1 (25) |  |  | 1 (25) |
|  | 13 |  |  | 1 (4) |  |  |  | 1 (4) |
|  | 14 |  |  | 1 (7) |  |  |  | 1 (7) |
|  | 15 |  |  |  | 1 (4) |  |  | 1 (4) |
|  | 20 |  |  | 1 (9) |  |  |  | 1 (9) |
| C |  | **2 (14)** | **3 (20)** | **2 (11)** | **3 (15)** | **1 (5)** | **11 (65)** | **5 (35)** |
|  | 1 | 1 (6) | 1 (6) | 1 (6) | 1 (5) | 1 (5) |  | 1 (6) |
|  | 4 |  |  | 1 (5) | 1 (4) |  |  | 1 (5) |
|  | 5 |  | 1 (5) |  |  |  |  | 1 (5) |
|  | 12 |  | 1 (9) |  | 1 (6) |  |  | 1 (11) |
|  | 16 | 1 (8) |  |  |  |  |  | 1 (8) |
| G |  | **4 (23)** | **3 (17)** | **2 (11)** | **2 (17)** | **1 (5)** | **12 (73)** | **7 (50)** |
|  | 1 | 1 (6) | 1 (6) | 1 (5) | 1 (4) | 1(5) |  | 1 (6) |
|  | 4 | 1 (4) |  |  |  |  |  | 1 (4) |
|  | 6 | 1 (3) |  |  |  |  |  | 1 (3) |
|  | 10 |  | 1 (8) |  |  |  |  | 1 (8) |
|  | 16 |  |  |  | 1 (13) |  |  | 1 (13) |
|  | 17 | 1 (10) | 1 (3) |  |  |  |  | 1 (10) |
|  | 18 |  |  | 1 (6) |  |  |  | 1 (6) |
| P |  | **7 (177)** | **4 (99)** | **3 (34)** | **3 (39)** | **0** | **15 (349)** | **7 (177)** |
|  | 4 | 1 (3) | 1 (3) | 1 (3) | 1 (3) |  |  | 1 (3) |
|  | 10 | 1 (5) |  | 1 (5) |  |  |  | 1 (5) |
|  | 15 | 1 (4) | 1 (4) |  |  |  |  | 1 (4) |
|  | 16 | 1 (26) | 1 (16) | 1 (26) | 1 (10) |  |  | 1 (26) |
|  | 17 | 1 (19) |  |  |  |  |  | 1 (19) |
|  | 19 | 1 (76) | 1 (76) |  | 1 (26) |  |  | 1 (76) |
|  | 20 | 1 (44) |  |  |  |  |  | 1 (44) |
| S |  | **1 (**0**3)** | **0** | **0** | **1 (4)** | **0** | **2 (7)** | **1 (4)** |
|  | 4 | 1 (3) |  |  | 1 (4) |  |  | 1 (4) |
| Total |  | **15 (224)** | **14 (152)** | **9 (71)** | **9 (90)** | **2 (10)** | **49 (547)** | **20 (327)** |
|  | 1 | 1 (6) | 1 (6) | 1 (6) | 1 (6) | 1 (6) |  | 1 (8) |
|  | 2 | 1 (4) | 1 (3) |  | 1 (3) | 1 (4) |  | 1 (6) |
|  | 3 | 1 (4) | 1 (5) |  | 1 (5) |  |  | 1 (7) |
|  | 4 | 1 (4) | 01 (4) | 1 (5) | 01 (4) |  |  | 1 (8) |
|  | 5 | 1 (9) | 1 (9) |  |  |  |  | 1 (9) |
|  | 6 | 1 (3) | 1 (3) |  |  |  |  | 1 (3) |
|  | 7 | 1 (3) | 1 (3) |  |  |  |  | 1 (3) |
|  | 8 | 1 (15) |  |  |  |  |  | 1 (15) |
|  | 9 | 1 (3) | 1 (3) | 1 (3) |  |  |  | 1 (6) |
|  | 10 | 1 (4) | 1 (8) | 1 (5) |  |  |  | 1 (8) |
|  | 11 |  |  |  | 1 (4) |  |  | 1 (4) |
|  | 12 |  | 1 (9) |  | 1 (25) |  |  | 1 (27) |
|  | 13 |  |  | 1 (4) |  |  |  | 1 (4) |
|  | 14 |  |  | 1 (7) |  |  |  | 1 (7) |
|  | 15 | 1 (4) | 01 (4) |  | 1 (4) |  |  | 1 (6) |
|  | 16 | 1 (26) | 1 (16) | 1 (26) | 1 (13) |  |  | 1 (60) |
|  | 17 | 1 (19) | 1 (3) |  |  |  |  | 1 (19) |
|  | 18 |  |  | 1 (6) |  |  |  | 1 (6) |
|  | 19 | 1 (76) | 1 (76) |  | 1 (26) |  |  | 1 (77) |
|  | 20 | 1 (44) |  | 1 (9) |  |  |  | 1 (44) |

^a^cCDE ID corresponds to that in Supplementary Table S1.

**Supplementary Table S5. List of 327 aCDEs comprising 20 cCDEs**. The order of the cCDEs is identical to that in Supplementary Table S1.

| **ID^a^** | **cCDE Name** | **Component aCDE Name** |
| --- | --- | --- |
| 1 | Patient_Demography_Composite_DE (8) | Patient_Age_Value_DE |
|  |  | Patient_Name_DE |
|  |  | Patient_Number_DE |
|  |  | Patient_Gender_Type_DE |
|  |  | Resident_Registration__Number_of_Patients_DE |
|  |  | Recording_Date_DE |
|  |  | Attending_Physician_Name_Specify_DE |
|  |  | Name_of_Recording_Doctor_DE |
| 2 | Past_History_Allergy_Dictionary_Composite_DE (6) | Past_History_Food_Allergy_Specify_DE |
|  |  | Past_History_Other_Allergy_Specify_DE |
|  |  | Past_History_Allergy_Type_Category_DE |
|  |  | Allergy_Yes_or_No_Indicator_DE |
|  |  | Past_History_Drug_Allergy_Specify_DE |
|  |  | Allergy_Specify_DE |
| 3 | Admission_and_Operation_Repeated_Composite_DE (7) | Admission_and_Operation_History_Yes_or_No_Indicator_DE |
|  |  | Admission_and_Operation_History_Specify_DE |
|  |  | Admission_and_Operation_Time_Specify_DE |
|  |  | Operation_and_Treatment_Date_DE |
|  |  | Operation_Others_Description_DE |
|  |  | Operation_and_Treatment_Name_DE |
|  |  | Admission_Department_Specify_DE |
| 4 | Chief_Complaint_Dictionary_Composite_DE (8) | Chief_Complaint_Name_DE |
|  |  | Chief_Complaint_Specify_DE |
|  |  | Chief_Diagnosis_Name_of_Postoperative_Specify_DE |
|  |  | Chief_Diagnosis_Name_of_Preoperative_Specify_DE |
|  |  | Chief_Symptom_and__Reason_Coming_to_Help_of_Chief_Complaint_Specify_DE |
|  |  | Duration_of_Chief_Complaint_Specify_DE |
|  |  | Onset_of_Chief_Complaint_DE |
|  |  | Others_of_Chief_Complaint_DE |
| 5 | Pain_Composite_DE (9) | Abdominal_Pain_Plus_or_Minus_Indicator_DE |
|  |  | Abdominal_Pain_Symptom_Specify_DE |
|  |  | Chest_Pain_Plus_or_Minus_Indicator_DE |
|  |  | Chest_Pain_Symptom_Specify_DE |
|  |  | Frequency_of_Pain_Evaluation_Specify__DE |
|  |  | Instrument_of_Pain_Evaluation_Specify__DE |
|  |  | Intensity_of_Pain_Evaluation_Specify__DE |
|  |  | Intervention_of_Pain_Evaluation_Specify__DE |
|  |  | Pain_Yes_or_No_Indicator_DE |
| 6 | Medicine_List_Composite_DE (3) | Other_Take_Medicine__Specify_DE |
|  |  | Take_Medicine_of_the_Origin_Specify_DE |
|  |  | Take_Medicine_Yes_or_No_Indicator_DE |
| 7 | BMI_Composite_DE (3) | BMI(Body_Mass_Index)__Measurement__DE |
|  |  | Weight(Kg)_Measurement_DE |
|  |  | Height(cm)__Measurement_DE |
| 8 | Patient_Evaluation_Composite_DE (15) | Patient_Age_Value_DE |
|  |  | Evaluation_of_Specific__Patients_Type_Category_DE |
|  |  | Infant_and_Adolescent_Family_Relations_Type_Category__DE |
|  |  | Infant_and_Adolescent_Intelligence_Type_Category__DE |
|  |  | Infant_and_Adolescent_Exercise_Development_Type_Category__DE |
|  |  | Infant_and_Adolescent_Growth_Development_(Height,Weight)_Type_Category_DE |
|  |  | Secondary_Sexual_Character_of_Adolescent_Type_Category_DE |
|  |  | Patients_Friendship_of_Adolescent_Type_Category_DE |
|  |  | The_Old_and_Infirm_Dressing_Type_Category_DE |
|  |  | The_Old_and_Infirm_Bathing_Type_Category_DE |
|  |  | The_Old_and_Infirm_Meal_Type_Category_DE |
|  |  | The_Old_and_Infirm_Movement_Type_Category_DE |
|  |  | The_Old_and_Infirm_Toilet_Use_Type_Category_DE |
|  |  | The_Old_and_Infirm_Urine_and_Feces_Control_Type_Category_DE |
|  |  | Infectious_Patient_Type_Category_DE |
| 9 | Past_History_Dictionary_Composite_DE (18) | Family_History__Type_Category_DE |
|  |  | Family_History_Description_DE |
|  |  | Family_History_Yes_or_No_Indicator_DE |
|  |  | Past_History_Type_Category_DE |
|  |  | Past_History_Description_DE |
|  |  | Past_History_Hepatitis_Type_Category_DE |
| 10 | Patient_Vital_Sign_Repeated_Composite_DE (8) | Blood_Pressure_of_Vital_Sign_Measurement_DE |
|  |  | Body_Temperature_of_Vital_Sign_Measurement_DE |
|  |  | Pulse_of_Vital_Sign_Measurement_DE |
|  |  | Respiratory_of_Vital_Sign_Measurement_DE |
|  |  | Vital_Sign_Type_Category_DE |
|  |  | Vital_Sign_Unstable_Specify_DE |
|  |  | Diastolic_Blood_Pressure__(mmHg)_Measurement_DE |
|  |  | Systolic_Blood_Pressure__(mmHg)_Measurement_DE |
| 11 | Current_Medication_List_Composite_DE (4) | Present_Taking_Drug_and__Taking_Medical_Treatment__Related_Matters_Specify_DE |
|  |  | Present_Taking_Drug_Name_Specify_DE |
|  |  | Present_Taking_Medicine_and__Taking_Medical_Treatment_Type_Category_DE |
|  |  | Medicine_and__Treatment_Follow_Up_Specify_DE |
| 12 | Examination_Finding_Dictionary_Composite_DE (27) | Conjunctiva_Status_Specify_DE |
|  |  | Neck_Vein_Status_Specify_DE |
|  |  | Sclera_Status_Specify_DE |
|  |  | Blood_Yes_or_No_Indicator_DE |
|  |  | Blue-Dot_Sign_Yes_or_No_Indicator_DE |
|  |  | Cremasteric_Reflex_Yes_or_No_Indicator_DE |
|  |  | CVA_Tenderness_Yes_or_No_Indicator_DE |
|  |  | Mass_Yes_or_No_Indicator_DE |
|  |  | Pitting_Edema_Yes_or_No_Indicator_DE |
|  |  | Examination_Finding_Specify_DE |
|  |  | Mass_Type_Category_DE |
|  |  | Pitting_Edema_Location__Category_DE |
|  |  | Sclera_Status_Category_DE |
|  |  | CT/SONO/MRI_Examination_Finding__Specify_DE |
|  |  | CT/SONO/MRI_Examination_Name_Specify_DE |
|  |  | CXR_Examination_Finding_Specify_DE |
|  |  | Eye_Status_Examination__Result___DE |
|  |  | Eye_Status_Examination__Specify__DE |
|  |  | Neck_Status_Examination__Result__DE |
|  |  | Neck_Status_Examination__Specify__DE |
|  |  | Neurological__Examination_Status_Specify_DE |
|  |  | Preadmission_Examination_Assessment_Specify_DE |
|  |  | Skin_Status_Examination_Result_DE |
|  |  | Skin_Status_Examination_Specify_DE |
|  |  | Throat_Status_Examination__Result___DE |
|  |  | Throat_Status_Examination__Specify__DE |
|  |  | HbsAg/Ab_Examination_DE |
| 13 | Treatment_Finding_Composite_DE (4) | Treatment_and_Progress_Other_Specify_DE |
|  |  | Treatment_Occurrence_DE |
|  |  | Treatment_Result_Category__DE |
|  |  | Treatment_Result_Specify_DE |
| 14 | Discharge_Medication_Dictionary_Composite_DE (7) | Medication_Dose_Frequency_DE |
|  |  | Medication_Occurrence_DE |
|  |  | Medication_Plan__Specify_DE |
|  |  | Medication_Prescription__Number_of_days_DE |
|  |  | Medication_Quantity__DE |
|  |  | Medication_Specify_DE |
|  |  | Medication_Unit_DE |
| 15 | Person_Weight_Change_Composite_DE (6) | Weight_Gain_Measurement_DE |
|  |  | Weight_Gain_Plus_or_Minus_Indicator_DE |
|  |  | Weight_Loss_Measurement_DE |
|  |  | Weight_Loss_Plus_or_Minus_Indicator_DE |
|  |  | Period_and_Type_of_Weight_Change__Specify__DE |
|  |  | Weight_Change_Gap_Specify__DE |
| 16 | Physical_Exam_Dictionary_Composite_DE (60) | Head_Exam_Other_comment_Specify_DE |
|  |  | Head_Status_Examination____DE |
|  |  | HEENT(Head__Ear__Eye__Nose_and_Throat)_Other_Status_Specify_DE |
|  |  | Chest/Lung_Heart_Sounds_Status_Specify_DE |
|  |  | Heart_Beat_Specify_DE |
|  |  | Heart_Rhythm_and__Murmur_Status_Category_DE |
|  |  | Heart_Sounds_Status_Specify_DE |
|  |  | Heart_Type_Category_DE |
|  |  | Past_Heart_Disease__Yes_or_No_Indicator_DE |
|  |  | Rectal_Examination_Yes_or_No_Indicator_DE |
|  |  | Rectal_Examination__Other_Specify_DE |
|  |  | Rectal_Examination_Result_DE |
|  |  | Genitourinary_Examination_Other_Specify_DE |
|  |  | Genitourinary_Result_DE |
|  |  | Genitourinary_Type_Category_DE |
|  |  | Back_&Extremity_P/Ex_Pitting_Edema__Yes_or_No_Indicator_DE |
|  |  | Back_and_Extremity_LOM_Yes_or_No_Indicator_DE |
|  |  | Back_and_Extremity_Other_Specify_DE |
|  |  | Back_and_Extremity_Result_DE |
|  |  | Mental_Status__Specify_DE |
|  |  | Mental_Status_Examination__Result__DE |
|  |  | Physical_Examination_Mental_Status__Category_DE |
|  |  | General_of_Physical_Examination_Specify_DE |
|  |  | HEENT_Physical_Examination_Palpable_Neck_Mass_Yes_or_No_Indicator_DE |
|  |  | Neck_Physical_Examination_Other_Specify_DE |
|  |  | Other_Physical_Examination_Specify_DE |
|  |  | Physical_Examination_Results_DE |
|  |  | Physical_Examination_Abnormal_Type_Category_DE |
|  |  | Physical_Examination_Mental_Status__Category_DE |
|  |  | Physical_Examination_Non_Specific_DE |
|  |  | Physical_Examination_Nutritional_and_Developement_Status_Category_DE |
|  |  | Physical_Examination_of_Abdomen_Other_Specify_DE |
|  |  | Physical_Examination_Type_Category_DE |
|  |  | Palpable_Cervical_L/N_Category_DE |
|  |  | Palpable_Cervical_L/N_Size_Measurement_DE |
|  |  | Abdomen_P/Ex__Flat_Specify_DE |
|  |  | Abdomen_P/Ex__Soft_Specify_DE |
|  |  | Abdomen_P/Ex_Palpation_of_Organ_Category_DE |
|  |  | Chest/Lung_P/Ex_Other__Specify_DE |
|  |  | Abdomen_Status_Description_DE |
|  |  | Abdomen_Status_Examination_Result_DE |
|  |  | Abdomen_Tenderness_Plus_or_Minus_Indicator_DE |
|  |  | Abdomen_Type_Category_DE |
|  |  | Chest/Lung_Breath_Sounds(BS)_Status_Specify_DE |
|  |  | Chest/Lung_Retraction,_Yes_or_No_Indicator_DE |
|  |  | Chest/Lung_Rhonchi,_Yes_or_No_Indicator_DE |
|  |  | Chest/Lung_Status_Type_Category_DE |
|  |  | Chest/Lung_Symmetric_Expansion,_Yes_or_No_Indicator_DE |
|  |  | Chest/Lung_Wheezing,_Yes_or_No_Indicator_DE |
|  |  | Chest_Status_Description_DE |
|  |  | Chest_Status_Examination__Result__DE |
|  |  | Chest_X-ray_Specify_DE |
|  |  | Atopy_Plus_or_Minus_Indicator_DE |
|  |  | Atopy_Specify_DE |
|  |  | DM(diabetes_mellitus)_Plus_or_Minus_Indicator_DE |
|  |  | DM(diabetes_mellitus)_Specify_DE |
|  |  | Hepatitis_Plus_or_Minus_Indicator_DE |
|  |  | Hepatitis_Specify_DE |
|  |  | HTN(hypertension)_Plus_or_Minus_Indicator_DE |
|  |  | HTN(hypertension)_Specify_DE |
| 17 | Social_History_Composite_DE (19) | Quit_Smoking_Period_DE |
|  |  | Smoking__Plus_or_Minus_Indicator_DE |
|  |  | Smoking__Specify_DE |
|  |  | Smoking_History_Per_Day_Cigarettes_Consumption_Count_DE |
|  |  | Smoking_History_Smoking_Period_(Year)_DE |
|  |  | Smoking_History_Type_Category_DE |
|  |  | Drinking_Frequency_Specify_DE |
|  |  | Drinking_History_Alcohol_Type_Category_DE |
|  |  | Drinking_History_Month_Frequency__DE |
|  |  | Drinking_History_Once_Drinking_Capacity_DE |
|  |  | Drinking_History_Period_DE |
|  |  | Drinking_History_Type_Category_DE |
|  |  | Social_History_Description_DE |
|  |  | Social_History_Marital_Status__DE |
|  |  | Social_History_Occupation_Name_DE |
|  |  | Alcohol_History_Plus_or_Minus_Indicator_DE |
|  |  | Alcohol_History_Specify_DE |
|  |  | Alcohol_Measurement_DE |
|  |  | Alcohol_Type_Others_Specify_DE |
| 18 | Transfer_Discharge_Composite_DE (6) | When_Transfer_Discharge_Drug_Preparation__Specify_DE |
|  |  | When_Transfer_Discharge_Hospital_Name_and_Contacted_Physician_Specify_DE |
|  |  | When_Transfer_Discharge_Other_Preparation__Specify_DE |
|  |  | When_Transfer_Discharge_Preparation_Items_Type_Category_DE |
|  |  | When_Transfer_Discharge_Transfer_Hospital_Selection_Method_Specify_DE |
|  |  | When_Transfer_Discharge_Transfer_Reason_Specify_DE |
| 19 | General_System_Dictionary_Composite_DE (77) | Anemic_Conjunctiva,_Plus_Minus_Indicator_DE |
|  |  | Anorexia_Plus_or_Minus_Indicator_DE |
|  |  | Arrythmia_Plus_or_Minus_Indicator_DE |
|  |  | Arthralgia_Plus_or_Minus_Indicator_DE |
|  |  | Asthenia_Universalis_Plus_or_Minus_Indicator_DE |
|  |  | Chill_Plus_or_Minus_Indicator_DE |
|  |  | Constipation_Plus_or_Minus_Indicator_DE |
|  |  | Cough_Plus_or_Minus_Indicator_DE |
|  |  | Cyanosis_Plus_or_Minus_Indicator_DE |
|  |  | Diarrhea_Plus_or_Minus_Indicator_DE |
|  |  | Dizziness_Plus_or_Minus_Indicator_DE |
|  |  | Dyspnea_Plus_or_Minus_Indicator_DE |
|  |  | Fatigue_Plus_or_Minus_Indicator_DE |
|  |  | Fever_Plus_or_Minus_Indicator_DE |
|  |  | Gastric_Ulcer_Plus_or_Minus_Indicator_DE |
|  |  | Headache_Plus_or_Minus_Indicator_DE |
|  |  | Hematemesis_Plus_or_Minus_Indicator_DE |
|  |  | Hematochezia_Plus_or_Minus_Indicator_DE |
|  |  | Icteric_Sclera,_Plus_Minus_Indicator_DE |
|  |  | Melena_Plus_or_Minus_Indicator_DE |
|  |  | Nausea_Plus_or_Minus_Indicator_DE |
|  |  | Palpitation_Plus_or_Minus_Indicator_DE |
|  |  | Paralysis_Plus_or_Minus_Indicator_DE |
|  |  | Reflex_Direct_of_Lt._Pupil_Plus_or_Minus_Indicator_DE |
|  |  | Reflex_direct_of_Rt._Pupil_Plus_or_Minus_Indicator_DE |
|  |  | Reflex_Indirect_of_Lt._Pupil_Plus_or_Minus_Indicator_DE |
|  |  | Reflex_Indirect_of_Rt._Pupil_Plus_or_Minus_Indicator_DE |
|  |  | Rhinorrhea_Plus_or_Minus_Indicator_DE |
|  |  | Seizure_Plus_or_Minus_Indicator_DE |
|  |  | Sensory_Change_Plus_or_Minus_Indicator__DE |
|  |  | Sore_Throat_Plus_or_Minus_Indicator_DE |
|  |  | Sputum_Plus_or_Minus_Indicator_DE |
|  |  | Tachypnea_Plus_or_Minus_Indicator_DE |
|  |  | Tinnitis_Plus_or_Minus_Indicator_DE |
|  |  | Tremor_Plus_or_Minus_Indicator_DE |
|  |  | Vomiting_Plus_or_Minus_Indicator_DE |
|  |  | Weakness_Plus_or_Minus_Indicator_DE |
|  |  | Anorexia_Symptom_Specify_DE |
|  |  | Arrythmia_Symptom_Specify_DE |
|  |  | Arthralgia_Symptom_Specify_DE |
|  |  | Chill_Symptom_Specify_DE |
|  |  | Constipation_Symptom_Specify_DE |
|  |  | Cough_Symptom_Specify__DE |
|  |  | Cyanosis_Symptom_Specify__DE |
|  |  | Diarrhea_Symptom_Specify_DE |
|  |  | Dyspnea_Symptom_Specify__DE |
|  |  | Fatigue_Symptom_Specify_DE |
|  |  | Fever_Symptom_Specify_DE |
|  |  | Headache_symptom_Specify_DE |
|  |  | Hematemesis_Symptom_Specify_DE |
|  |  | Hematochezia_Symptom_Specify_DE |
|  |  | Melena_Symptom_Specify_DE |
|  |  | Nausea_Symptom_Specify_DE |
|  |  | Palpitation_Symptom_Specify_DE |
|  |  | Paralysis_Symptom_Specify_DE |
|  |  | Rhinorrhea_Symptom_Specify_DE |
|  |  | Seizure_Symptom_Specify_DE |
|  |  | Sensory_Change_Symptom_Specify_DE |
|  |  | Sore_Throat_Symptom_Specify_DE |
|  |  | Sputum_Symptom_Specify_DE |
|  |  | Tachypnea_Symptom_Specify_DE |
|  |  | Tinnitis_Symptom_Specify__DE |
|  |  | Tremor_Symptom_Specify_DE |
|  |  | Vomiting_Symptom_Specify_DE |
|  |  | Weakness_Symptom_Specify_DE |
|  |  | Asthenia_Universalis_Specify_DE |
|  |  | Dizziness_Symptom_DE |
|  |  | Gastric_Ulcer_Specify_DE |
|  |  | Gastrointestinal_Status_Specify_DE |
|  |  | Neuromuscular_Status_Specify_DE |
|  |  | Respiratory_Status_Specify_DE |
|  |  | Spine_Status_Specify_DE |
|  |  | Tongue_Status_Category_DE |
|  |  | Cardiovascular_Status_Specify_DE |
|  |  | G.C.S._(Glasgow_Coma_Scale)_Eye__Score_DE |
|  |  | G.C.S._(Glasgow_Coma_Scale)_Motor__Score_DE |
|  |  | G.C.S._(Glasgow_Coma_Scale)_Verbal__Score_DE |
| 20 | Lab_Result_Dictionary_Composite_DE (44) | Urine_Bilirubin_Test_DE |
|  |  | Urine_Clarity_Test_DE |
|  |  | Urine_Color_Test_DE |
|  |  | Urine_Culture_Test_DE |
|  |  | Urine_Cytology_Test_DE |
|  |  | Urine_Occult_Blood_Test_DE |
|  |  | Urine_PH_Measurement_DE |
|  |  | Urine_Protein_Measurement_DE |
|  |  | Cardiac_Profile_Test_DE |
|  |  | Image__Test_Result_and_Other_Data_Specify_DE |
|  |  | Leukocyte_Esterase_Test_DE |
|  |  | Nitrite_Test_DE |
|  |  | Phenyl_Ketonuria__Test_DE |
|  |  | Pulmonary_Function__Test_Specify_DE |
|  |  | Urobilinogen_Test_DE |
|  |  | Venereal_Disease__Research_Laboratory_Test_DE |
|  |  | Activated_Partial__Thromboplastin_Time__Measurement_DE |
|  |  | Alanine_Transaminase_Measurement_DE |
|  |  | Albumin_Measurement_DE |
|  |  | Alkaline_Phosphatase_Measurement_DE |
|  |  | Amylase_Measurement_DE |
|  |  | Aspartate__Transaminase_Measurement_DE |
|  |  | Blood_Urea_Nitrogen_Measurement_DE |
|  |  | Calcium_Measurement_DE |
|  |  | Chloride_Measurement_DE |
|  |  | C-reactive_Protein_Measurement_DE |
|  |  | Creatine_Kinase_MB_Measurement_DE |
|  |  | Creatinine_Measurement_DE |
|  |  | Direct_Bilirubin_Measurement_DE |
|  |  | Electrolyte_Measurement_DE |
|  |  | Glomerular_Filtration__Rate__Measurement_DE |
|  |  | Kalium_Measurement_DE |
|  |  | Lactate_Dehydrogenase_Measurement_DE |
|  |  | Magnesium_Measurement_DE |
|  |  | Natrium_Measurement_DE |
|  |  | Prothrombin_Time__Measurement_DE |
|  |  | Pupil_Size(R,L)_Measurement_DE |
|  |  | Rt_pupil__Measurement_DE |
|  |  | T._Cholesterol_Measurement_DE |
|  |  | Total_Bilirubin_Measurement_DE |
|  |  | Total_Protein_Measurement_DE |
|  |  | Uric_Acid_Measurement_DE |
|  |  | Left_Pupil_Size_Measurement(mm)_DE |
|  |  | Right_Pupil_Size_Measurement(mm)_DE |

^a^cCDE ID corresponds to that in Supplementary Table S1.

**Supplementary Table S6. Distribution of aCDEs and cCDEs extracted from 14 FHIR resources of FHIR bulk sample data**. List of unique 75 aCDEs comprising by 28 cCDEs from 238 aCDEs. The absence of *repeated* cCDE for some FHIR resources means that the configuration of aCDEs has been changed for each data.

| **ID^a^**  **No.** | #1 | #2 | #3 | #4 | #5 | #6 | #7 | #8 | #9 | #10 | #11 | #12 | #13 | #14 |
| --- | --- | --- | --- | --- | --- | --- | --- | --- | --- | --- | --- | --- | --- | --- |
|  | **2(13)** | **4(15)** | **5(13)** | **2(13)** | **3(9)** | **4(15)** | **1(4)** | **3(14)** | **1(4)** | **3(14)** | **5(18)** | **4(15)** | **8(29)** | **3(13)** |
| 1 | 1(4) | 1(4) |  | 1(4) | 1(4) | 1(4) |  |  | 1(4) | 1(4) | 1(4) | 1(4) | 1(4) | 1(4) |
| 2 |  | 1(2) | 1(2) |  |  | 1(2) |  |  |  |  |  | 1(2) |  | 1(2) |
| 3 |  | 1(5) |  |  |  |  |  |  |  |  |  |  |  |  |
| 4 |  | 1(4) |  |  |  |  |  |  |  |  | 1(4) |  |  |  |
| 5 |  |  | 1(4) |  |  |  |  |  |  |  |  |  | 1(4) |  |
| 6 |  |  | 1(2) |  |  |  |  |  |  |  |  | 1(2) |  | 1(2) |
| 7 |  |  | 1(2) |  |  |  |  |  |  |  |  |  |  |  |
| 8 |  |  | 1(3) |  |  |  |  |  |  |  |  |  |  |  |
| 9 |  |  |  |  | 1(2) |  |  |  |  | 1(2) |  |  |  |  |
| 10 |  |  |  |  |  | 1(3) |  | 1(3) |  |  |  |  |  |  |
| 11 |  |  |  |  |  |  |  | 1(4) |  |  | 1(4) |  | 1(4) |  |
| 12 |  |  |  |  |  |  |  | 1(7) |  |  |  |  |  |  |
| 13 |  |  |  |  |  |  |  |  |  |  | 1(4) |  | 1(4) |  |
| 14 |  |  |  |  |  |  |  |  |  |  |  | 1(5) |  |  |
| 15 |  |  |  |  |  |  |  |  |  |  |  |  | 1(3) |  |
| 16 |  |  |  |  |  |  |  |  |  |  |  |  | 1(2) |  |
| 17 |  |  |  |  |  |  |  |  |  |  |  |  | 1(6) |  |
| 18 |  |  |  |  |  |  |  |  |  |  |  |  | 1(2) |  |
| 19**^b^** | 1(13) |  |  |  |  |  |  |  |  |  |  |  |  |  |
| 20**^b^** |  |  |  | 1(13) |  |  |  |  |  |  |  |  |  |  |
| 21**^b^** |  |  |  |  |  | 1(15) |  |  |  |  |  |  |  |  |
| 22**^b^** |  |  |  |  |  |  | 1(4) |  |  |  |  |  |  |  |
| 23**^b^** |  |  |  |  |  |  |  |  |  | 1(14) |  |  |  |  |
| 24**^b^** |  |  |  |  |  |  |  |  |  |  |  | 1(15) |  |  |
| 25**^b^** |  |  |  |  |  |  |  |  |  |  |  |  |  | 1(13) |
| 26 |  | 1(6) |  |  |  |  |  |  |  |  |  |  |  |  |
| 27 |  |  |  |  | 1(5) |  |  |  |  |  |  |  |  |  |
| 28 |  |  |  |  |  |  |  |  |  |  | 1(6) |  |  |  |

^a^ FHIR Resource ID corresponds to that in Table 4.

**^b^** All CDEs of each FHIR resource become component aCDEs of *Repeated* cCDE.

**Supplementary Table S7. List of 75 aCDEs comprising by 28 cCDEs**. The order of the cCDEs is identical to that in Supplementary Table S4.

| **ID^a^** | **cCDE Name** | **Component aCDE Name** |
| --- | --- | --- |
| 1 | Code_Composite_DE | Code_System_DE |
|  |  | Code_DE |
|  |  | Code_Display_DE |
|  |  | Code_Name_Text_DE |
| 2 | Period_Composite_DE | Start_DE |
|  |  | End_DE |
| 3 | Activity_Detail_ Composite_DE | Code_System_DE |
|  |  | Code_DE |
|  |  | Code_Display_DE |
|  |  | Code_Name_Text_DE |
|  |  | Activity_Status_DE |
| 4 | Category_Code_Composite_DE | Category_Code_System_DE |
|  |  | Category_Code_DE |
|  |  | Category_Code_Display_DE |
|  |  | Category_Code_Name_Text_DE |
| 5 | Information_Code_Comspite_DE | Information_Sequence_DE |
|  |  | Code_System_DE |
|  |  | Code_DE |
|  |  | Value_reference_DE |
| 6 | Diagmosis_Comspite_DE | Sequence_DE |
|  |  | Value_reference_DE |
| 7 | Item_Encounter_Comspite_DE | Sequence_DE |
|  |  | Encounter_Reference_DE |
| 8 | Total_Value_Comspite_DE | Total_Value_Text_DE |
|  |  | Code_System_DE |
|  |  | Code_DE |
| 9 | Result_Display_Comspite_DE | Result_Rerence_DE |
|  |  | Result_Display_Text_DE |
| 10 | Reason_Code_Comspite_DE | Code_System_DE |
|  |  | Code_DE |
|  |  | Code_Display_DE |
| 11 | Instance_Item_Comspite_DE | UID_DE |
|  |  | Number_DE |
|  |  | sopClass_Name_DE |
|  |  | Instance_Title_DE |
| 12 | Series_Comspite_DE | UID_DE |
|  |  | Number_DE |
|  |  | Modality_Text_DE |
|  |  | Number_Instance_DE |
|  |  | Body_Site_DE |
|  |  | Start_Text_DE |
|  |  | Instance_Text_DE |
| 13 | Value_Quantity_Comspite_DE | Value_DE |
|  |  | Unit_DE |
|  |  | Code_System_DE |
|  |  | Code_DE |
| 14 | Address_Simple Comspite_DE | Address_Line_DE |
|  |  | Address_City_DE |
|  |  | Postal_Code_DE |
|  |  | Address_State_DE |
|  |  | Country_DE |
| 15 | Person_Name_Comspite_DE | Use_Name_DE |
|  |  | Family_Name_DE |
|  |  | Given_Name_DE |
| 16 | Status_Comspite_DE | Status_DE |
|  |  | Status_Text_DE |
| 17 | Address_Comspite_DE _ | Use_Address_DE |
|  |  | Address_Line_DE |
|  |  | Address_City_DE |
|  |  | Postal_Code_DE |
|  |  | Address_State_DE |
|  |  | Country_DE |
| 18 | URL_Comspite_DE _ | URL_Text_DE |
|  |  | Extended_Text_DE |
| 19 | AllergyIntolerance_FHIR_Repeat_Comspite_DE | Resource_Type_DE |
|  |  | ID_DE |
|  |  | Clinical_Status_DE |
|  |  | Verification_Status_DE |
|  |  | Type_DE |
|  |  | Category_Text_DE |
|  |  | Criticality_DE |
|  |  | Code_System_DE |
|  |  | Code_DE |
|  |  | Code_Display_DE |
|  |  | Code_Name_Text_DE |
|  |  | Condition_List_Text_DE |
|  |  | Patient_Reference_DE |
|  |  | Asserated_Date_DE |
| 20 | Claim_FHIR_Repeat_Comspite_DE | Resource_Type_DE |
|  |  | ID_DE |
|  |  | Clinical_Status_DE |
|  |  | Verification_Status_DE |
|  |  | Category_Text_DE |
|  |  | Criticality_DE |
|  |  | Code_System_DE |
|  |  | Code_DE |
|  |  | Code_Display_DE |
|  |  | Code_Name_Text_DE |
|  |  | Subject_Reference_DE |
|  |  | Context_DE |
|  |  | Onset_DateTime_DE |
|  |  | Abatement_DateTime_DE |
|  |  | Asserated_Date_DE |
| 21 | DiagnosticReport_FHIR_Repeat_Comspite_DE | Resource_Type_DE |
|  |  | ID_DE |
|  |  | Clinical_Status_DE |
|  |  | Class_Code_DE |
|  |  | Code_System_DE |
|  |  | Code_DE |
|  |  | Code_Display_DE |
|  |  | Code_Name_Text_DE |
|  |  | Subject_DE |
|  |  | Start_DE |
|  |  | End_DE |
|  |  | Reason_Code_System_DE |
|  |  | Reason_Code_DE |
|  |  | Reason_Code_Display_DE |
|  |  | Service_Provider_DE |
| 22 | Encounter_FHIR_Repeat_Comspite_DE | Resource_Type_DE |
|  |  | ID_DE |
|  |  | Clinical_Status_DE |
|  |  | Description_DE |
| 23 | Immunization_FHIR_Repeat_Comspite_DE | Resource_Type_DE |
|  |  | ID_DE |
|  |  | Clinical_Status_DE |
|  |  | Intent_Text_DE |
|  |  | Code_System_DE |
|  |  | Code_DE |
|  |  | Code_Display_DE |
|  |  | Code_Name_Text_DE |
|  |  | Subject_Reference_DE |
|  |  | Context_DE |
|  |  | Authored_On_DE |
|  |  | Reason_Reference_DE |
|  |  | Dosage_Instruction_squence_DE |
|  |  | Dosage_Instruction_Boolean_DE |
| 24 | Observation_FHIR_Repeat_Comspite_DE | Resource_Type_DE |
|  |  | ID_DE |
|  |  | Identifier_System_Text_DE |
|  |  | Identifier_Value_DE |
|  |  | Code_System_DE |
|  |  | Code_DE |
|  |  | Code_Display_DE |
|  |  | Code_Name_Text_DE |
|  |  | Person_Name_DE |
|  |  | Telecom_Terminology_DE |
|  |  | Telecom_Value_DE |
|  |  | Address_Line_DE |
|  |  | Address_City_DE |
|  |  | Postal_Code_DE |
|  |  | Address_State_DE |
|  |  | Country_DE |
| 25 | Procedure_FHIR_Repeat_Comspite_DE | Resource_Type_DE |
|  |  | ID_DE |
|  |  | Clinical_Status_DE |
|  |  | Code_System_DE |
|  |  | Code_DE |
|  |  | Code_Display_DE |
|  |  | Code_Name_Text_DE |
|  |  | Subject_Reference_DE |
|  |  | Context_DE |
|  |  | Start_DE |
|  |  | End_DE |
|  |  | Reason_Code_DE |
|  |  | Reason_Code_Display_DE |
| 26 | Care_Related_Condition_Dictionary_DE | Code_System_DE |
|  |  | Code_DE |
|  |  | Code_Display_DE |
|  |  | Code_Name_Text_DE |
|  |  | Condition_List_Text_DE |
|  |  | Activity_List_Text_DE |
| 27 | Diagnostic_Related_Result_Dictionary_DE | Code_System_DE |
|  |  | Code_DE |
|  |  | Code_Display_DE |
|  |  | Code_Name_Text_DE |
|  |  | Diagnostic_Related_Result_List_DE |
| 28 | Category_Related_Result_Dictionary_DE | Category_Code_System_DE |
|  |  | Category_Code_DE |
|  |  | Category_Code_Display_DE |
|  |  | Code_System_DE |
|  |  | Code_DE |
|  |  | Code_Display_DE |

**Supplementary Table S8. List of categorized MIMIC-III database in which matched by aCDE, cCDE and constraints**. Six tables are related *hybrid* and *variable* aCDE (23%), four tables are related *dictionary* cCDE (15%), and all tables are related to *required* constraints.

| Category Name | Table Name | No. of Columns | aCDE | cCDE | Constraint |
| --- | --- | --- | --- | --- | --- |
| Hospital Database (14) | ADMISSION | 19 |  |  | Required |
|  | CALLOUT | 24 |  |  | Required |
|  | CPTEVENTS | 12 | Hybrid, Variable |  | Required, Operated |
|  | D_LABITEMS | 6 |  | Dictionary | Required |
|  | DIAGNOSES_ICD | 5 | Variable |  | Required |
|  | DRGCODE | 8 |  |  | Required |
|  | ICUSTAYS | 12 |  |  | Required |
|  | LABEVENTS | 9 | Hybrid, Variable |  | Required |
|  | MICROBIOLOGYEVENTS | 16 | Hybrid |  | Required |
|  | NOTEEVENTS | 11 |  |  | Required |
|  | PRESECIPTIONS | 19 |  |  | Required, Operated |
|  | PROCEDURES_ICD | 5 | Variable |  | Required |
|  | SERVICES | 6 |  |  | Required |
|  | TRANSFERS | 13 |  |  | Required |
| Online definitions (3) | D_CPT | 9 |  | Dictionary | Required |
|  | D_ICD_DIAGNOSES | 4 |  | Dictionary | Required |
|  | D_ICD_PROCEDURES | 4 |  | Dictionary | Required |
| CareValue and Metaversion ICU databases (9) | CAREGIVERS | 4 |  |  | Required |
|  | CHARTEVENTS | 15 | Hybrid |  | Required |
|  | D_ITEMS | 10 |  |  | Required |
|  | DATETIMEEVENTS | 14 |  |  | Required |
|  | INPUTEVENTS_CV | 22 |  |  | Required |
|  | INPUTEVENTS_MV | 31 |  |  | Required |
|  | OUTPUTEVENTS | 13 |  |  | Required |
|  | PATIENTS | 8 |  |  | Required |
|  | PROCEDURES_MV | 25 |  |  | Required |

**Supplementary Table S9. List the detail elements of MIMIC-III database, which were matched to our proposed DE types.** *Hybrid* aCDEs in four tables, *variable* aCDE in four tables, *operated* constraint in two tables.

| Table Name | Hybrid (4) | | | Variable (4) | Operated (2) | | |
| --- | --- | --- | --- | --- | --- | --- | --- |
|  | **Items^#^** | Exmple1 | Exmple2 |  | Items | Exmple1 | Exmple2 |
| CPTEVENTS | **CPT_CD** | Temporary codes | 99254 |  |  |  |  |
|  | CPT_NUMBER |  | 99254 | CPT_NUMBER^a^ | CPT_NUMBER |  |  |
|  | CPT_SUFFIX | T |  |  |  |  |  |
| LABEVENTS | **VALUE** | NORMAL | 90.6 |  |  |  |  |
|  | VALUENUM |  | 90.6 |  |  |  |  |
|  | VALUEUOM |  | % |  |  |  |  |
|  |  |  |  | ITEMID^b^ |  |  |  |
| MICROBIOLOGYEVENTS | **DILUTION_TEXT** | <=4 |  |  |  |  |  |
|  | DILUTION_COMPARISON | <= |  |  |  |  |  |
|  | DILUTION_VALUE | 4 |  |  |  |  |  |
| CHARTEVENTS | **VALUE** | X-ray | 83 |  |  |  |  |
|  | VALUENUM |  | 83 |  |  |  |  |
|  | VALUEUOM |  | IU/L |  |  |  |  |
| DIAGNOSES_ICD |  |  |  | ICD9_CODE ^c^ |  |  |  |
| PROCEDURES_ICD |  |  |  | ICD9_CODE ^d^ |  |  |  |
| PRESECIPTIONS |  |  |  |  | PROD_STRENGTH | 25mcg/0.5mL Vial | 10mg Tablet |
|  |  |  |  |  | DOSE_VAL_RX ^e^ | 0.5 | 20 |
|  |  |  |  |  | DOSE_UNIT_RX | mL | mg |
|  |  |  |  |  | FORM_VAL_DISP | 1 | 2 |
|  |  |  |  |  | FORM_UNIT_DISP | VIAL | TAB |

^#^ The hybrid aCDE is that the item name is in bold

^a^ Variable aCDE fornula: D_CPT.mincodeinsubsection <= CPTEVENTS.cpt_number <= D_CPT.maxcodeinsubsection

^b^ Variable aCDE fornula: LABEVENTS.ITEMID == D_LABITEMS.ITEMID

^c^ Variable aCDE fornula: DIAGNOSES_ICD.ICD9_CODE == D_ICD_DIAGNOSES.ICD9_CODE

^d^ Variable aCDE fornula: PROCEDURES_ICD.ICD9_CODE == D_ICD_PROCEDURES.ICD9_CODE

^e^ Calculated aCDE formula: PRESECIPTIONS.dose_val_rx (PRESECIPTIONS.dose_unit_rx) = PRESECIPTIONS.prod_strength * PRESECIPTIONS.form_val_disp (PRESECIPTIONS.form_unit_disp)
